# Phase separation of RNF214 promotes the progression of hepatocellular carcinoma

**DOI:** 10.1038/s41419-024-06869-2

**Published:** 2024-07-05

**Authors:** Zheng-Jie He, Ke He, Song-Wang Cai, Rui Zhang, Zhong-Bao Shao, Sheng-Te Wang, Xiao-Peng Li, Yan-Chi Li, Wei-Jing Liu, You-Qing Zhu, Shi-Jie Zeng, Yu-Bin Su, Zhi Shi

**Affiliations:** 1 Cancer Minimally Invasive Therapies Centre, Guangdong Second Provincial General Hospital, Jinan University, Guangzhou, Guangdong 510632 China; 2 Department of Cell Biology & Institute of Biomedicine, Guangdong Provincial Biotechnology & Engineering Technology Research Center, Guangdong Provincial Key Laboratory of Bioengineering Medicine, Genomic Medicine Engineering Research Center of Ministry of Education, MOE Key Laboratory of Tumor Molecular Biology, National Engineering Research Center of Genetic Medicine, State Key Laboratory of Bioactive Molecules and Druggability Assessment, College of Life Science and Technology, Jinan University, Guangzhou, Guangdong 510632 China; 3 Department of Surgery, The First Affiliated Hospital of Jinan University, Guangzhou, Guangdong 510632 China

**Keywords:** Tumour biomarkers, Predictive markers, Tumour biomarkers, Oncogenesis, Mechanisms of disease

## Abstract

Hepatocellular carcinoma (HCC) is one of the most common malignant tumors, and the expression and function of an uncharacterized protein RNF214 in HCC are still unknown. Phase separation has recently been observed to participate in the progression of HCC. In this study, we investigated the expression, function, and phase separation of RNF214 in HCC. We found that RNF214 was highly expressed in HCC and associated with poor prognosis. RNF214 functioned as an oncogene to promote the proliferation, migration, and metastasis of HCC. Mechanically, RNF214 underwent phase separation, and the coiled-coil (CC) domain of RNF214 mediated its phase separation. Furthermore, the CC domain was necessary for the oncogenic function of RNF214 in HCC. Taken together, our data favored that phase separation of RNF214 promoted the progression of HCC. RNF214 may be a potential biomarker and therapeutic target for HCC.

## Introduction

Phase separation refers to the formation of membrane-free organelles or biomolecular aggregates or liquid droplets in cells [1]. The process involves the movement of biomolecules from a uniform environment to a more concentrated phase, resulting in the formation of both a less crowded and a more crowded phase [2]. The condensed droplets formed by phase separation have liquid properties including sphericity, fusion, and fission [3]. The formation of different subcellular membraneless compartments, such as stress granules, bodies of Cajal, nucleoli, splicing spots, and processing bodies, relies on phase separation [4]. Phase separation can be triggered by the weak affinity and nonspecific interactions, drove by intrinsically disordered regions found in proteins participated in the formation of phase-separated aggregates [5]. Multivalent interactions, facilitated by intrinsically disordered regions and low-complexity regions, serve as the governing factor for the formation of phase separation aggregates within cells. Phase separation is primarily facilitated by proteins and nucleic acids, specifically DNA and RNA, which serve as the principal constituents and regulators [6]. Various mechanisms tightly regulate the protein phase separation, which can be influenced by physical factors like pH, temperature, ion concentration, and osmotic pressure [7].

Approximately 90% of patients diagnosed with liver cancer are attributed to hepatocellular carcinoma (HCC) [8, 9]. HCC is one of the most common human malignancies worldwide and it is predicted to reach over one million cases by 2025 [10]. Risk factors of HCC comprise infection of hepatitis B virus (HBV), cirrhosis related to alcohol abuse, diabetes, and obesity, etc. [11]. Despite the significant recent advancements in systemic treatments for HCC, the prognosis for HCC remains discouraging [12]. Therefore, elucidating of the mechanistic basis of HCC initiation and development is crucial for the therapeutic advantage of this disease.

In light of the escalating evidence suggesting a correlation between aberrant phase separation and cancer, research on the interplay between HCC and phase separation has been notably sparse [4]. As the research on phase separation expands, an increasing number of websites and datasets related to this phenomenon are becoming available. By integrating bioinformatics analysis with extensive cancer databases, a growing number of proteins, including RNF214, are gradually coming into focus. RNF214, known as RING finger protein 214, is recognized as a member of the RING finger protein family due to its RING domain structure. The human RNF214, located on chromosome 11q13.1, comprises one CC domain and one RING finger domain and is composed of 703 amino acids. Despite the limited research on RNF214, its RING finger domain suggests that it functions as an E3 ubiquitin ligase, potentially participating in cellular processes such as signal transduction, protein degradation, and cellular stress responses [13].

This study aims to investigate the expression and role of RNF214 in HCC. Our findings revealed that RNF214 functions as an oncogenic protein, driving HCC proliferation, migration, and metastasis through phase separation.

## Materials and methods

### Animal models of HCC

All animal experimental procedures were approved by the Institutional Animal Care and Use Committee of Jinan University (Approval Number 20211116-02) and were in accordance with the NIH Guide for the Care and Use of Laboratory Animals. Four-week-old female BALB/c nude mice were divided at random into groups. The laboratory technician was blinded to the group allocation of the mice throughout the experimental process. These mice were obtained from GemPharmatech (Nanjing, China). The given cells were injected into the mice in 100 μL of Dulbecco’s Modified Eagle Medium (DMEM) containing 2 × 10^6^ cells, either under both shoulders or through the lateral tail vein (*n* = 5 mice). Tumor volume was calculated using the formula: 1/2 (length × width^2^). No animals were excluded from the analysis due to ethical concerns. The sample size for each group was determined based on previous studies [14]. After administering anesthesia to the mice, the weights of the tumors and lungs were measured. The identification of cancer tissue and normal tissue was done using the staining method of hematoxylin and eosin, which was described earlier in ref. [14].

### Human samples

Forty cases of primary HCC tissues and non-cancer tissues were from patients who received surgery at the First Affiliated Hospital of Jinan University. The experimental protocols of human subjects were approved by the Institutional Review Board (IRB) committee of Jinan University (Approval Number JNUKY-2022-092). Patients or their relatives provided signed informed consent.

### Cell lines and reagents

Human embryonic kidney cell line HEK293T, HCC cell lines SK-HEP-1, MHCC97H, MHCC97L, Huh7, Hep-3B, HepG2, PLC/PRF/5, SNU387 (from the Cell Bank of the Chinese Academy of Sciences), SNU449 (from the American Type Culture Collection), and the non-malignant liver cell line MIHA (from Dr. J.R. Chowdhury, Albert Einstein College of Medicine, NY, USA) were cultured in DMEM supplemented with 10% fetal bovine serum (purchased from Thermo Fisher Scientific, Waltham, MA, USA), 100 ng/mL streptomycin, and 100 U/mL penicillin at 37 °C with 5% CO_2_ in a humidified incubator. The cells were free of mycoplasma contamination.

Reagents and antibodies included in the study were as follows: PEG8000, sorbitol, NaCl, sucrose, and other regents were sourced from Sangon Biotech (Shanghai, China). Polyetherimide (PEI, #23966-1) was purchased from Polysciences (Warrington, PA, USA). Anti-RNF214 antibody (#202826-T38) was purchased from Sino Biological (Beijing, China). Anti-GFP antibody (#D110008-0200) and anti-GST antibody (#D190101-0200) were purchased from Sangon Biotech (Shanghai, China). Anti-β-actin antibody (#CL594-66009) was sourced from Proteintech (Wuhan, China). Anti-Vinculin antibody (#A01207) was provided from BOSTER (Wuhan, China).

### Plasmids and lentivirus infection

For the expression of proteins in mammalian cells, plasmids were constructed using Gateway Cloning Technology, with pDEST27, pCDH-Neo-Venus/DEST, and pET-59-EGFP/DEST serving as the destination vectors for the generation of the corresponding RNF214 constructs. The full-length RNF214 protein, consisting of 703 amino acid residues, was utilized as a template (Gene ID: 257160). The resulting truncated constructs included segments spanning amino acids 1–206, 207–410, 411–657, and 658–703. Additionally, constructs representing the full-length N-terminal portion (1–410) and the C-terminal portion (411–703) were developed. A construct containing the CC domain (220–379) was also created, as well as a construct with the CC domain deleted (∆CC), which resulted in the deletion of 160 amino acids.

For the construction of stable cell lines, CRISPR/Cas9 technology was also employed. The two sgRNAs targeting human RNF214 (sgRNA1: 5′-cataaactagaagattccgg-3′, sgRNA2: 5′-tcagacgaaggtctcccaga-3′) were cloned into the vector LentiCRISPR V2. RNF214 was cloned either in full length or truncated form into pCDH-Neo-Venus/DEST vectors according to previously described methods [15]. HEK293T cells were used to package the lentivirus, which was then collected from the supernatant of the medium. Cells were infected with the lentivirus to establish stable cell lines and were then selected using puromycin or G418. Monoclonal cells were selected by limiting dilution, and knockout efficiency was verified by western blotting and genome sequencing as previously mentioned [16].

### Protein expression and purification

Full-length and truncations of RNF214 were cloned into pET-59-EGFP/DEST vector which was constructed by inserting an EGFP element into the pET-59-DEST vector. Proteins in *Escherichia coli* BL21 (DE3) cells were induced by adding 0.1 mM isopropyl-β-D-thiogalactoside at 26 °C for 6 h. The cells were then lysed using a sonication lysis solution, which contained 50 mM Tris-HCl (pH 7.5), 50 mM NaCl, 0.1 mg/mL lysozyme, 5% glycerol, and 1 mM phenylmethylsulfonyl fluoride. Sonication was performed after that. After centrifugation, the lysates were incubated with Ni-IDA Sepharose 6FF (Sangon Biotech, #C600029) at 4 °C for 4 h or overnight. The resin was washed four times with washing buffer (20 mM Tris-HCl, pH 8.0, 0.5 M NaCl, and 20 mM imidazole). The resin was then eluted with elution buffer (20 mM Tris-HCl, pH 8.0, 0.5 M NaCl, and 300 mM imidazole).

To confirm the correct insertion of the target gene into the expression vector, plasmid DNA was sequenced in its entirety. The molecular weight of the purified protein was estimated by SDS–PAGE followed by Coomassie staining and compared with a protein ladder to ensure it matched the expected size. The identity of the recombinant protein was further confirmed by western blot using antibodies specific to the GFP tag or the protein itself. After undergoing SDS–PAGE analysis, the proteins were confirmed and divided into aliquots for storage at −80 °C.

### GST pull-down assay

HEK293T cells were transfected with 1 μg each of plasmids encoding for GST-fusion or Venus-fusion proteins using PEI. Following a 48-h incubation at 37 °C in a CO_2_ incubator, cells were treated with trypsin to detach them, collected, and lysed on ice. The lysate was clarified by centrifugation at 4 °C, and the supernatant was mixed with 50 μL of GST-tag Purification Resin (Beyotime, #P2251). The mixture was incubated at 4 °C overnight with continuous rotation. After incubation, the supernatant was discarded, and the resin was washed eight times with ice-cold cell lysis buffer to remove unbound proteins. The bound proteins were then eluted by adding 2× loading buffer, mixed, and heated at 100 °C for 10 min. The supernatant was collected and used for a western blot to analyze the presence of the GST or Venus-tagged proteins.

### Fluorescence recovery after photobleaching

Purified proteins were prepared at a concentration of 1 μM and mixed with 10% PEG8000. Cells were then observed under the Zeiss LSM 900 confocal microscope (Oberkochen, Germany). Puncta were photobleached with a 488 nm laser at 100% power. Images were acquired once per second after bleaching.

### Western blot

Proteins were isolated on 10–12% SDS–PAGE gels, loaded onto polyvinylidene difluoride membranes, blocked with 5% skimmed milk, and probed with the corresponding primary antibody followed by horseradish peroxidase-linked secondary antibody. After three washes with TBST buffer (150 mM NaCl, 10 mM Tris-HCl, and 0.1% (v/v) Tween 20, pH 7.6), signals were examined by the ChemiDoc XRS chemiluminescent gel imaging system (Analytik Jena, Jena, Germany).

### Cell proliferation assay

Cell proliferation was examined by methyl thiazolyl tetrazolium (MTT) assay and colony formation assays. For MTT assay, cells were cultured in 96-well plates for the indicated times. Then 0.5 mg/ml MTT was added to each well. Formazan crystals were dissoluted in 50 μL DMSO and absorbance was recorded at 570 nm after incubation for 4 h. For colony formation assay, cells were cultured in 12-well plates for 2 weeks, followed by fixation in 4% paraformaldehyde and stained for 10 min by 0.1% crystal violet. The colonies were photographed and counted under the microscope.

### Cell migration assay

Cell migration was detected by wound healing and transwell assays. For the wound healing assay, cells at 90% confluence in six-well plates were scratched by a 200 μL pipet tip and washed twice using PBS. After 24 h, the cells were permitted to migrate in a serum-free culture medium and the appearance of the wounds was monitored and documented. For transwell assay, cells were seeded in the insert with serum-free culture medium. Each insert was then placed in the lower chamber of 24-well plates containing a culture medium consisting of 10% fetal bovine serum. After cultured for 24 h, cells on the upper side of the membrane were removed, while the cells penetrating the lower side were photographed. Cells were then immersed in 4% paraformaldehyde to fix them for 10 min and stained using 0.1% crystal violet for a further 10 min.

### Immunofluorescence assay

Slides were used to culture cells, which were then treated either with or without NaCl. After removal of the medium, cells were fixed by 4% paraformaldehyde for 15 min and then washed three times by PBS for 5 min each. Cells were permeabilized by 0.3% Triton X-100 and then blocked for 1 h using 5% BSA at room temperature. Afterward, cells were immersed in their primary antibody for a whole night at 4 °C and washed three times in PBS. After the addition of fluorescent secondary antibodies, cells were incubated in the dark for 1 h, followed by three washes using PBS. After incubation with 10 min of DAPI, the slides were washed three times by PBS and sealed with anti-fluorescence quencher, and the images were captured with a confocal microscope (Zeiss LSM 900).

### Immunohistochemistry assay

Tissue slides were deparaffinized using xylene and graded ethanol. For antigen retrieval, the slides were boiled in 10 mM citrate buffer for 5 min. After cooling two times at ambient temperature, slides were incubated for 15 min using 0.05% Triton X-100 followed by quenching with 3% H_2_O_2_ for the same duration. A blocking step was performed using 3% BSA for 30 min. Subsequently, the slides were washed by PBS and incubated with the primary antibody overnight at 4 °C. Following this, the slides were immersed in secondary antibody for 30 min and stained with DAB and hematoxylin. The protein expression was quantified as previously described [17]. Briefly, immunohistochemistry scores were performed by calculating the percentage of positive cells multiplied with the intensity score. Intensity was scored using the following scoring system: 0 for no staining, 1 for mild (pale yellow), 2 for moderate (yellowish brown), and 3 for strong (brown). An immunohistochemical score of ≥50 was considered positive.

### Statistical analysis

Statistics were described as mean ± standard deviation (SD). GraphPad Prism 8 and SPSS Statistics 24 were used for statistics analysis. The Student’s *t*-test and one-way ANOVA were used to compare two groups and multiple groups, respectively. The Kaplan–Meier survival analysis was used to describe overall survival. The independent predictors of prognosis were investigated by Cox proportional hazards regression analysis. The Fisher exact test or Chi-square test was applied for categorical data. The critical value for statistical significance was assessed as *p* < 0.05.

## Results

### RNF214 is highly expressed in HCC and correlated with poor prognosis

**Fig. 1:**
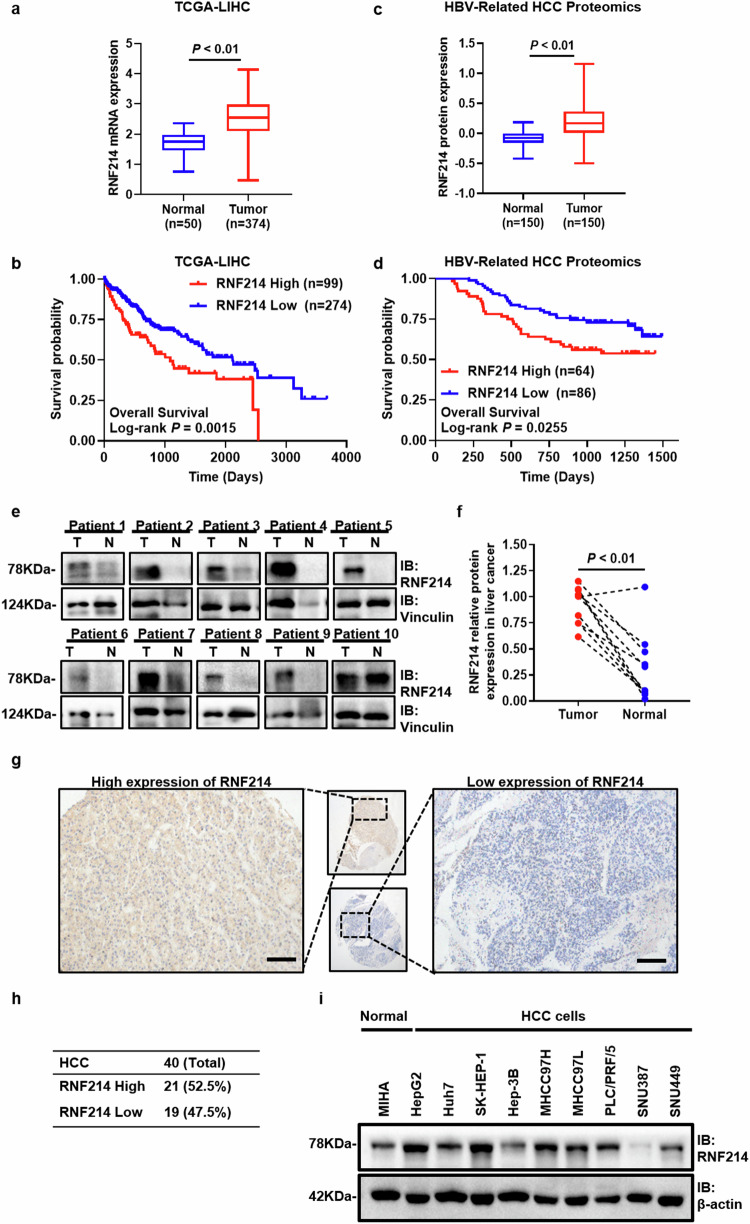
RNF214 is overexpressed in HCC and correlates with unfavorable prognosis. **a** The mRNA levels of RNF214 between HCC and normal in the TCGA database were shown (normal *n* = 50, tumor *n* = 374, two-tailed Student’s *t*-test). **b** The overall survival curves for HCC patient groups with high and low RNF214 expression from TCGA databases were shown (*n* = 373, Kaplan–Meier survival curve analysis). **c** The protein levels of RNF214 between HCC and normal in HBV-related HCC proteome databases were shown (*n* = 150, two-tailed Student’s *t*-test). **d** The overall survival curves for HCC patient groups with high and low RNF214 expression from HBV-related HCC proteome databases were shown (*n* = 150, Kaplan–Meier survival curve analysis). **e**, **f** The protein levels and quantification of RNF214 in ten sets of HCC tissues and their paired normal tissues were displayed (*n* = 10, two-tailed Student’s *t*-test). **g**, **h** The protein levels and quantification of RNF214 were measured by IHC including 40 HCC patients, and representative images were presented. The scale bar is 100 μm. **i** The protein levels of RNF214 in normal hepatocyte MIHA and HCC cells (HepG2, Huh7, SK-HEP-1, Hep-3B, MHCC97H, MHCC97L, PLC/PRF/5, SNU387, and SNU449) were shown. The *n* represents the number in each group. The bar graph data are presented as the mean ± SD. The *p* value < 0.05 indicates statistical significance.

To investigate the relationship between HCC and phase separation, we compiled a dataset of proteins associated with phase separation along with transcriptomic and proteomic datasets of HCC. Following an intersection analysis and validation of these datasets, we identified RNF214 as a potential protein that may be highly expressed in HCC and related to phase separation. To validate the expression and function of RNF214 in HCC, we searched the Cancer Genome Atlas database (TCGA) and HBV-related HCC proteome databases [18]. The results discovered the protein and mRNA levels of RNF214 in HCC tissues were remarkably elevated compared with normal tissues. Furthermore, based on Kaplan–Meier analysis, the survival rate of HCC patients with elevated RNF214 levels in both databases was significantly inferior to those with lower RNF214 expression (Fig. 1a–d). Subsequently, RNF214 protein expression was observed in ten sets of HCC tissues and matching normal tissues. According to the results, the levels of RNF214 protein expression were significantly elevated in HCC tissues compared to normal tissues (Fig. 1e, f). Furthermore, immunohistochemistry results of the tissue array including 40 HCCs showed that RNF214 was highly expressed in 52.5% of HCC tissues (Fig. 1g, h). Additional examination of HBV-related HCC proteome database revealed a positive association between RNF214 expression and alpha-fetoprotein (AFP) level (Table S1). Cox regression analysis was performed to conduct both univariate and multivariate survival prediction models to explore the independent prognostic factors of HCC patients. The data presented that high expression of RNF214, tumor recurrence, and abnormal AFP levels were independent risk factors for HCC prognosis (Table S2). In addition, we also observed the expression of RNF214 in HCC cells. In comparison to normal liver cells MIHA, RNF214 protein expression in multiple HCC cells was higher (Fig. 1i). These data suggest that RNF214 exhibits high expression levels associated with inferior prognosis in HCC.

### Knockout of RNF214 suppresses HCC proliferation

**Fig. 2:**
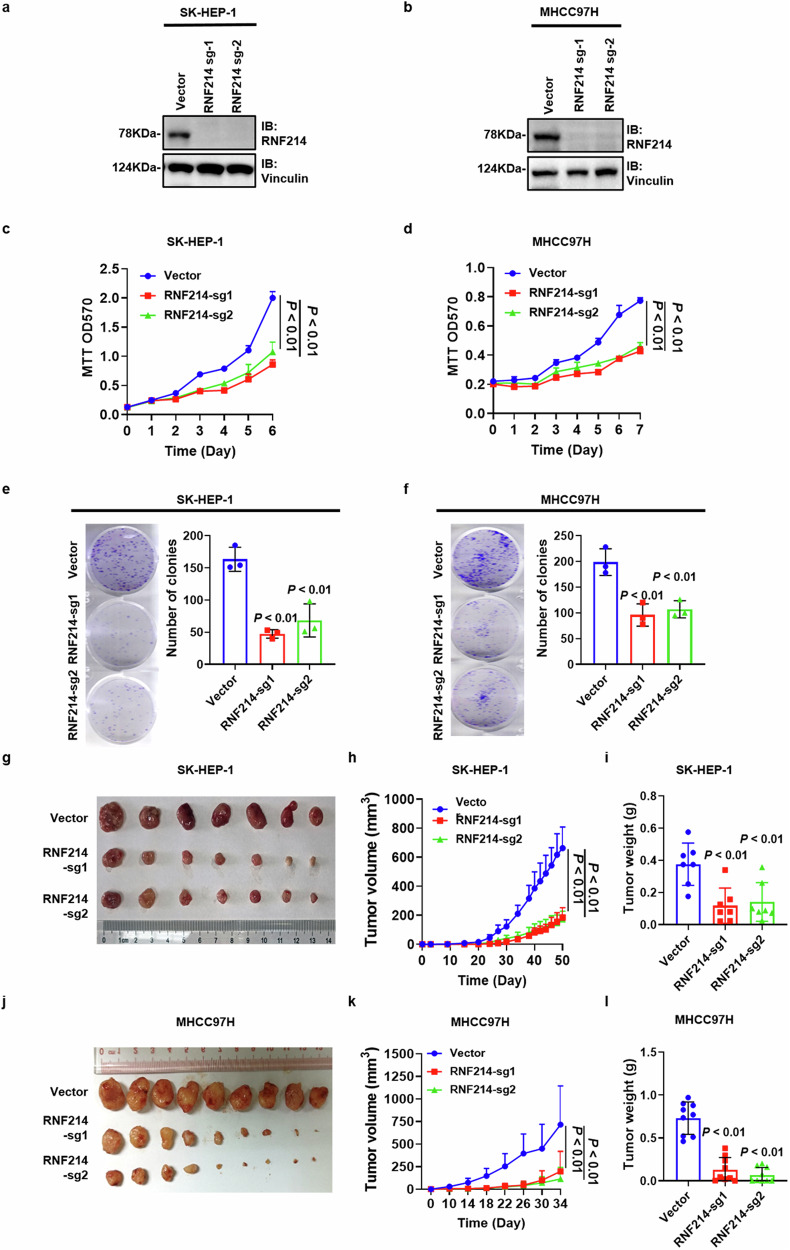
Knockout of RNF214 inhibits HCC proliferation. **a**, **b** The protein levels in the corresponding cells were displayed. **c**, **d** Cell number growth of the selected cells was estimated with MTT assay (*n* = 3, one-way ANOVA). **e**, **f** The typical images and quantitative data of the displayed colony numbers were calculated by colony formation assay (*n* = 3, one-way ANOVA). **g**–**l** The indicated subcutaneous tumors were shown as well as the quantification of tumor weight and volume (*n* = 5 for BALB/c nude mice, one-way ANOVA). The *n* represents the number of biologically independent experiments in each group. The bar graph data are presented as the mean ± SD. The *p* value < 0.05 indicates statistical significance.

To explore the potential functions of RNF214 within HCC cells, two cell lines SK-HEP-1 and MHCC97H with high endogenous RNF214 expression were selected for knockout. RNF214 knockout monoclonal cells were generated utilizing two individual sgRNA sequences in both SK-HEP-1 and MHCC97H cells (Fig. S1a, b). Knockout of RNF214 was verified by western blot (Fig. 2a, b) and genome sequencing (Fig. S1c, d). MTT assay was performed to determine whether knockout of RNF214 suppresses the proliferation of HCC cells, and the results showed that knockout of RNF214 suppressed the growth of SK-HEP-1 and MHCC97H cells lines (Fig. 2c, d). Moreover, colony formation assay results also showed that depletion of RNF214 decreased the number of clones in both SK-HEP-1 and MHCC97H cell lines (Fig. 2e, f). Furthermore, the results of subcutaneous xenograft in nude mice revealed that the absence of RNF214 reduced the size and mass of the implanted tumors, which were generated by both SK-HEP-1 and MHCC97H cell lines (Fig. 2g–l). The evidence suggests that inhibition of HCC proliferation is observed when RNF214 is knocked out.

### Knockout of RNF214 inhibits HCC migration and metastasis

**Fig. 3:**
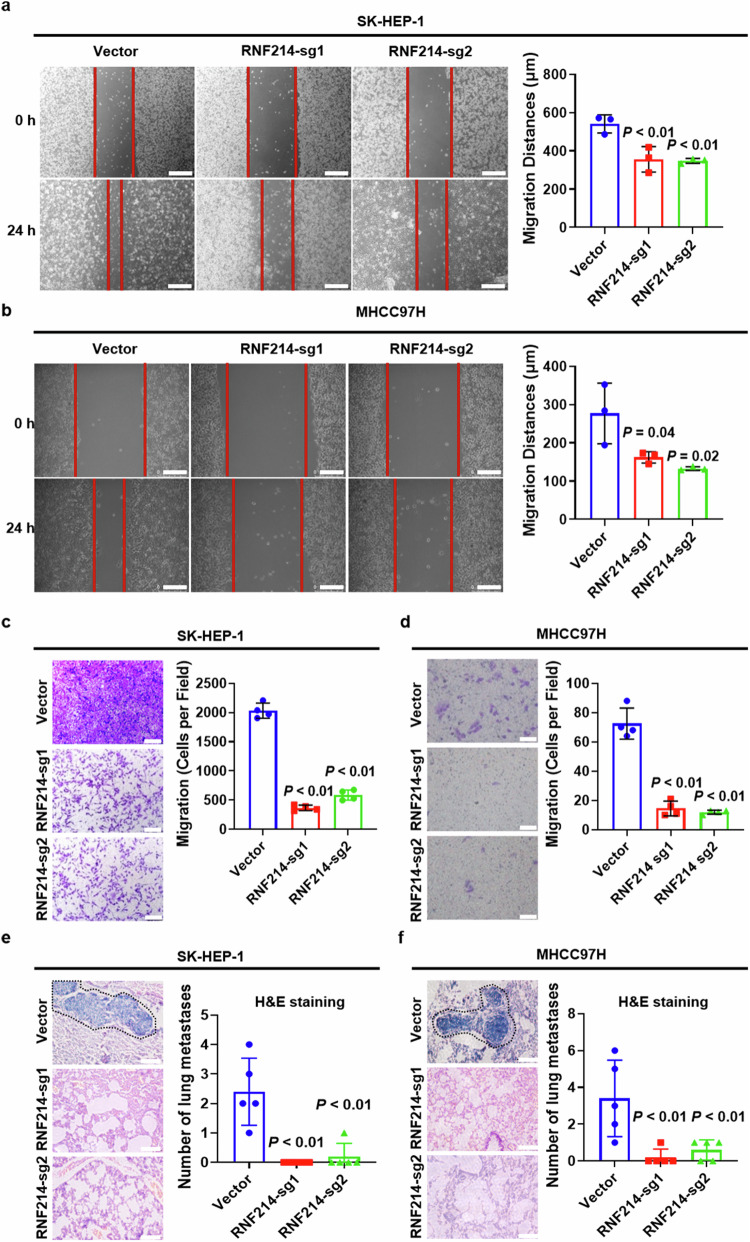
Knockout of RNF214 inhibits HCC migration and metastasis. **a**, **b** Graphical illustration and quantification of cell migration were taken via wound healing test. The scale bar is 250 μm (*n* = 3, one-way ANOVA). **c**, **d** Graphical illustration and quantification of cell migration were assessed by transwell assay. Scale bar is 100 μm (*n* = 4, one-way ANOVA). **e**, **f** Graphical illustration and quantitative analysis of pulmonary metastasis in the selected lung tissue sections were performed with hematoxylin and eosin staining. Scale bar is 100 μm (*n* = 5, one-way ANOVA). The *n* represents the number of biologically independent experiments in each group. The bar graph data are presented as the mean ± SD. The *p* value < 0.05 indicates statistical significance.

To explore whether knockout of RNF214 affected the migration of HCC cells, wound healing assays were performed, and the results showed that knockout of RNF214 diminished the migration distance of both SK-HEP-1 and MHCC97H cells (Fig. 3a, b). Similarly, the results of transwell assay showed that knockout of RNF214 attenuated the migration ability of both SK-HEP-1 and MHCC97H cells (Fig. 3c, d). To further investigate the effect of RNF214 knockout on HCC cell metastasis, the models of lung metastasis were established by injecting cells into the tail vein of nude mice. The results revealed that the absence of RNF214 reduced the amount of lung metastatic tumors generated by SK-HEP-1 and MHCC97H cells (Fig. 3e, f). These data demonstrate that knockout of RNF214 inhibits HCC migration and metastasis.

### RNF214 undergoes phase separation

**Fig. 4:**
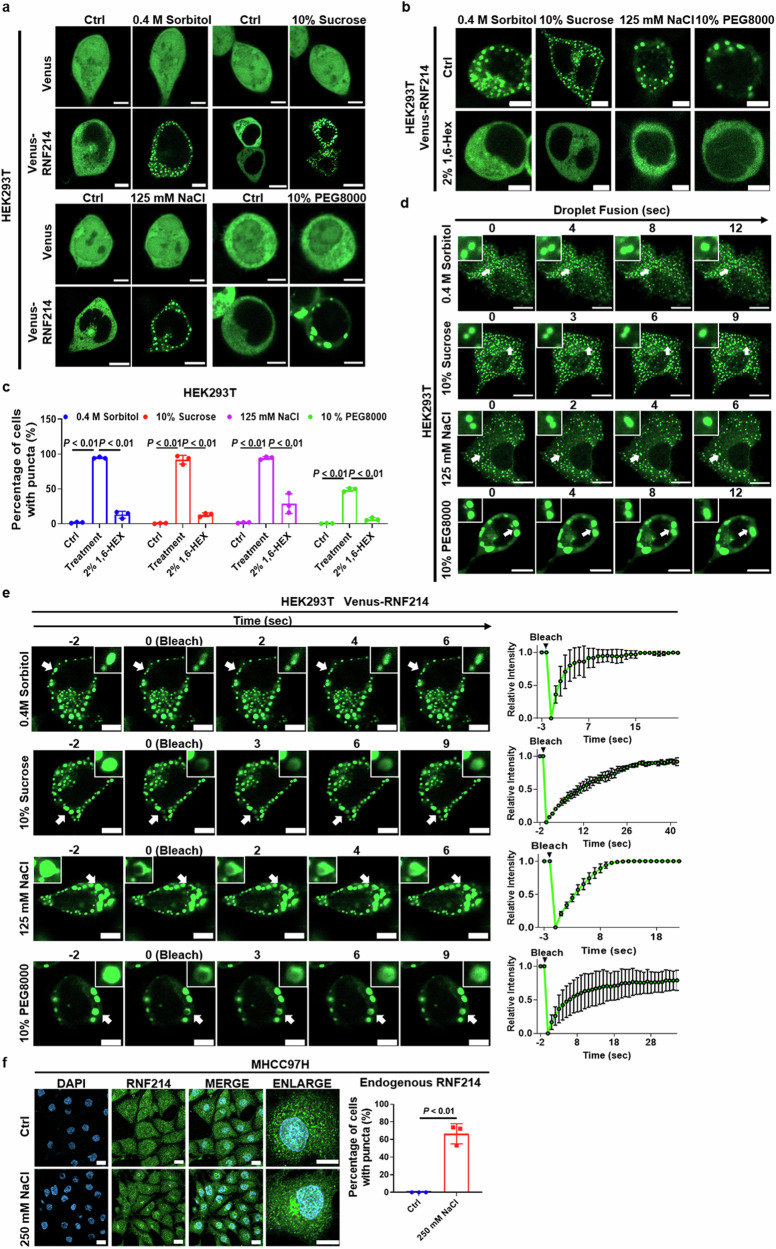
RNF214 undergoes phase separation. **a**–**c** Venus and Venus-RNF214 transfected HEK293T cells were viewed and quantified after treatment with 0.4 M sorbitol, 10% sucrose, 10% PEG8000, or 125 mM NaCl for 10 min and 2% 1,6-hexanediol for another 3 min (*n* = 3, at least 100 cells, two-tailed Student’s *t*-test). **d** Observation of the puncta fusion. **e** Observation of the puncta after the fluorescence bleaching. Scale bar is 10 μm. **f** The endogenous RNF214 in MHCC97H cells was observed and quantified with immunofluorescence assay after treatment with 250 mM NaCl for 10 min. Blue represented DAPI and green represented endogenous RNF214 (*n* = 3, at least 100 cells, two-tailed Student’s *t*-test). Low magnification picture scale bar are 50 μm, and high magnification picture scale bar are 10 μm. The *n* represents the number of random fields of view in each group. The bar graph data are presented as the mean ± SD. The *p* value < 0.05 indicates statistical significance.

Recent reports have demonstrated that phase separation plays a critical role in regulating tumorigenesis [19–22]. Furthermore, osmotic stress represents one of the stress types capable of inducing phase separation, wherein high salt concentration, high sucrose concentration, sorbitol addition, and polyethylene glycol addition disrupt cellular osmotic balance, thereby mimicking osmotic stress in physiological or pathological contexts. To test whether RNF214 undergoes phase separation, the fluorescent protein Venus and Venus-RNF214 vectors were constructed. Then HEK293T cells were transfected and treated with 0.4 M sorbitol, 10% sucrose, 10% PEG8000, or 125 mM NaCl for 10 min. The results showed that Venus-RNF214 not Venus formed the speckle-like puncta in HEK293T cells under all these treatments (Fig. 4a). After being treated with 2% 1,6-hexanediol for another 3 min, the puncta disappeared (Fig. 4b, c). Moreover, the puncta could fuse and gradually recover after fluorescence bleaching (Fig. 4d, e). To further examine whether endogenous RNF214 undergoes phase separation, an immunofluorescence assay was used to detect the endogenous RNF214 in MHCC97H cells, and the results showed that RNF214 also formed the puncta after being treated with 250 mM NaCl for 10 min (Fig. 4f). These results suggest that RNF214 can undergo phase separation.

### The CC domain of RNF214 mediates its phase separation

**Fig. 5:**
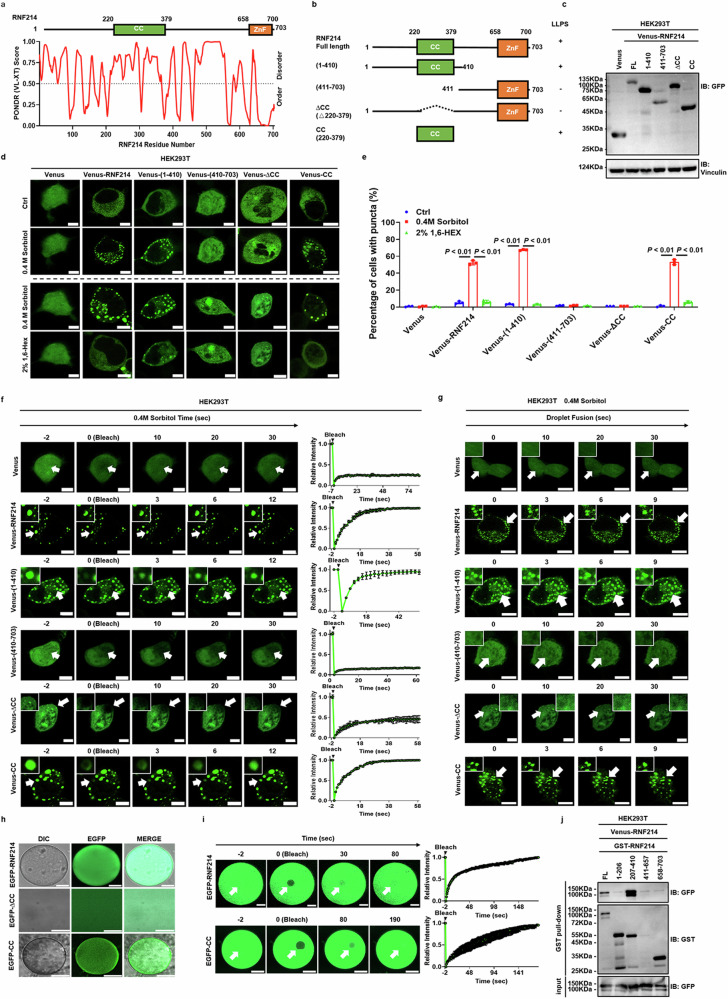
The CC domain of RNF214 mediates its phase separation*.* **a** RNF214 protein domain and protein disorder region predicted by PONDR were shown. **b** The schematic representation of RNF214 truncations was shown. **c** The protein levels in the indicated cells were shown. **d**, **e** HEK293T cells transfected with Venus, Venus-RNF214 full-length, and truncations were observed and quantified after being treated with 0.4 M sorbitol for 10 min and 2% 1,6-hexanediol for another 3 min (*n* = 3, at least 100 cells, two-tailed Student’s *t*-test). The *n* represents the number of random fields of view in each group. The bar graph data are presented as the mean ± SD. The *p* value < 0.05 indicates statistical significance. **f** Observation of the puncta fusion. **g** Observation of the puncta after the fluorescence bleaching. Scale bar = 10 μm. **h**, **i** Observation of the puncta of purified proteins (EGFP-RNF214, EGFP-CC, and EGFP-ΔCC) after the fluorescence bleaching in vitro. Scale bar = 20 μm. **j** HEK293T cells were transfected with Venus-RNF214 and full-length or truncated forms of GST-tagged RNF214 (amino acids 1–206, 207–410, 411–657, and 658–703), followed by GST pull-down assays.

To identify which region mediates phase separation of RNF214, the truncations of RNF214 expressing vectors were constructed based on the domain and predicted disordered region (Fig. 5a). The N-terminal (amino acids 1–410) and C-terminal (amino acids 411–703) constructs were used for preliminary screening to identify the regions within RNF214 where phase separation occurs. Further confirmation of the domain responsible for phase separation was conducted using the CC domain (amino acids 220–379) and the ∆CC (from which 160 amino acids were deleted) constructs (Fig. 5b). HEK293T cells were transfected and verified by western blot (Fig. 5c). Similar to Veneus-RNF214, coiled-coil (CC) domain (Venus-CC) not deletion of CC domain (Venus-ΔCC) could form the puncta (Fig. 5d, e), and the puncta of Venus-CC could also fuse and gradually recover after fluorescence bleaching (Fig. 5f, g). In addition, the purified proteins of EGFP-RNF214 and EGFP-CC not EGFP-ΔCC also undergo phase separation and gradually recover after fluorescence bleaching in vitro (Fig. 5h, i). To further confirm whether the CC domain also mediates the phase separation of RNF214 formation in HCC cells, MHCC97H cells were transfected with Venus, Venus-RNF214, Venus-ΔCC, and Venus-CC expressing vectors and treated with 0.8 M sorbitol or 250 mM NaCl for 10 min. The results showed that both Venus-RNF214 and Venus-CC, but not Venus and Venus-ΔCC formed the puncta in MHCC97H cells under all these treatments. After being treated with 2% 1,6-hexanediol for another 3 min, the puncta also disappeared (Figs. S2a, b and S3a, b). Moreover, the puncta could also fuse and gradually recover after fluorescence bleaching (Figs. S2c, d and S3c, d). We also observed that under the same conditions, the punctum formed by Venus-CC were brighter and had a clearer outline compared to those formed by Venus-FL, and their morphology was more spherical in shape (Figs. 5, S3, S4). Additionally, the CC domain, which is commonly implicated in the formation of dimers or multimers, was investigated for its role in the oligomerization of RNF214. Utilizing a GST pull-down assay, we confirmed that RNF214 can form dimers through a truncated construct containing the CC domain (amino acids 207–410). Conversely, a truncated construct harboring the RING finger domain (amino acids 658–703) was incapable of forming dimers (Fig. 5j). These data indicate that the CC domain of RNF214 mediates its phase separation.

### The CC domain is essential for RNF214 to promote HCC proliferation

**Fig. 6:**
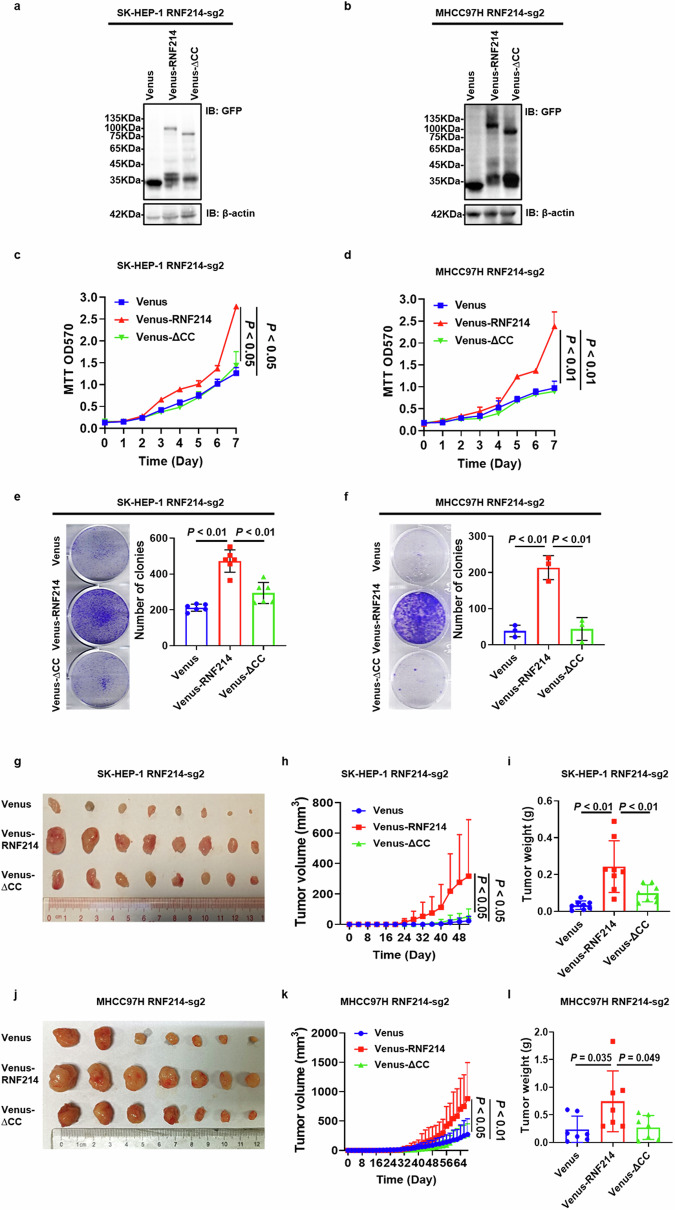
The CC domain is required for RNF214 to enhance HCC proliferation. **a**, **b** The protein levels in the selected cells were displayed. **c**, **d** Cell number growth of the selected cells was estimated with MTT assay (*n* = 3, one-way ANOVA). **e**, **f** The typical images and quantitative data of the shown cell colonies were measured by colony formation assay (*n* ≥ 3, one-way ANOVA). **g**–**l** Representative images of the displayed subcutaneous tumors as well as quantification of tumors weight and volume were demonstrated (*n* = 5 for BALB/c nude mice, one-way ANOVA). The *n* represents the number of biologically independent experiments in each group. The bar graph data are presented as the mean ± SD. The *p* value < 0.05 indicates statistical significance.

To investigate the impact of the CC domain on the influence of RNF214 on the growth of HCC cells, Venus, Venus-RNF214, and Venus-∆CC were reintroduced into RNF214-deficient HCC cells (SK-HEP-1 RNF214-sg2 and MHCC97H RNF214-sg2) via stable expression (Fig. 6a, b). MTT test results demonstrated that Venus-RNF214 promoted the growth of both SK-HEP-1 RNF214-sg2 and MHCC97H RNF214-sg2 cells more compared to Venus-∆CC (Fig. 6c, d). Moreover, the colony formation assay results also indicated that Venus-RNF214 increased the quantity of clones in both SK-HEP-1 RNF214-sg2 and MHCC97H RNF214-sg2 cells more significantly compared to Venus-∆CC (Fig. 6e, f). Furthermore, the subcutaneous xenograft model results in nude mice revealed that Venus-RNF214 expanded the size and mass of transplanted tumors generated by both SK-HEP-1 RNF214-sg2 and MHCC97H RNF214-sg2 cells more significantly compared to Venus-∆CC (Fig. 6g–l). These experimental results indicate that the CC region is essential for RNF214 to enhance HCC growth.

### The CC domain is essential for RNF214 to promote HCC migration and metastasis

**Fig. 7:**
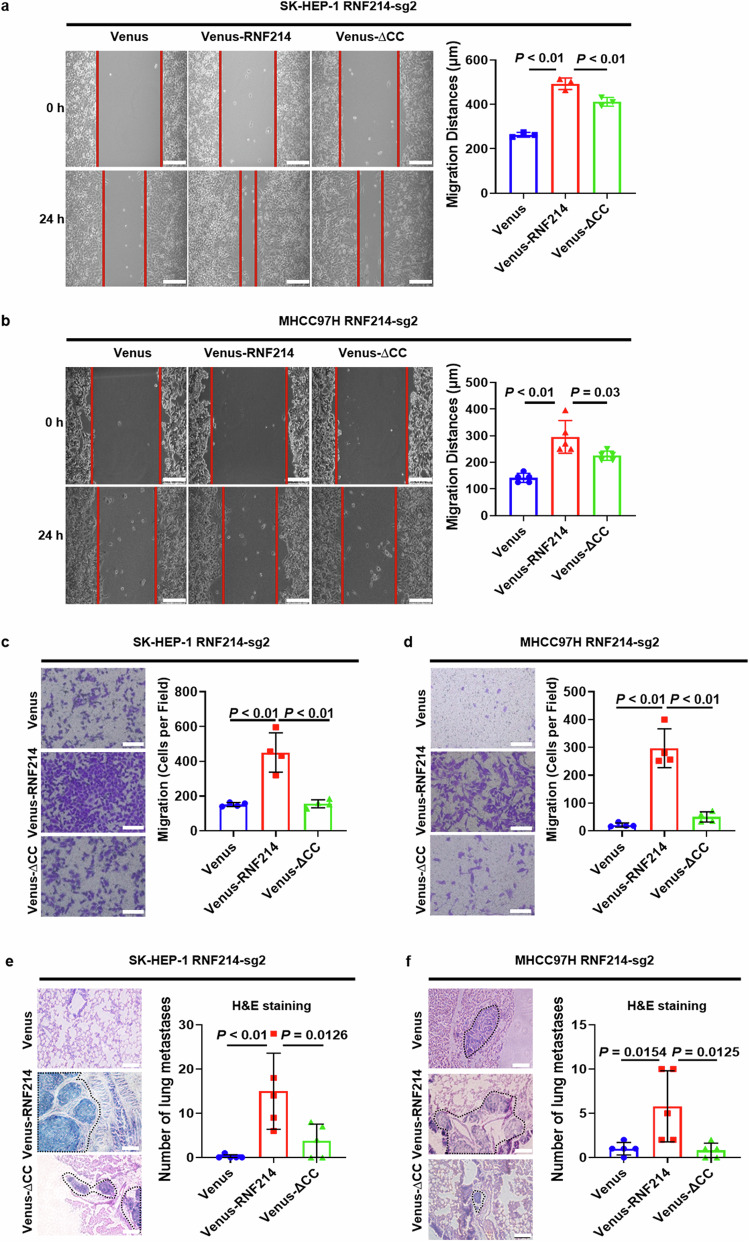
The CC domain is critical for RNF214 to enhance HCC migration and metastasis. **a**, **b** Representative illustrations and quantification of displayed cell migration were taken via wound healing test. Scale bar is 250 μm (*n* ≥ 3, one-way ANOVA). **c**, **d** Representative illustrations as well as quantification of the displayed cell migration were detected by transwell assay. Scale bar is 100 μm (*n* = 4, one-way ANOVA). **e**, **f** Representative illustrations and quantification of lung metastasis in the displayed lung sections were detected by hematoxylin and eosin staining, scale bars are 100 μm (*n* = 5, one-way ANOVA). The *n* represents the number of biologically independent experiments in each group. The bar graph data are presented as the mean ± SD. The *p* value < 0.05 indicates statistical significance.

To examine the function of the CC domain in the influence of RNF214 on the migration of HCC cells, wound healing assay was conducted. The results revealed that Venus-RNF214 resulted in a greater migration distance for both SK-HEP-1 RNF214-sg2 and MHCC97H RNF214-sg2 cells in comparison to Venus-∆CC (Fig. 7a, b). Similarly, the results of transwell assay suggested that Venus-RNF214 enhanced the migration speed of both SK-HEP-1 RNF214-sg2 and MHCC97H RNF214-sg2 cells more than Venus-∆CC (Fig. 7c, d). To further evaluate the influence of the CC domain on RNF214-mediated metastasis of HCC cells, we additionally performed the models of lung metastasis via tail vein injection in nude mice. According to the results, Venus-RNF214 increased the quantity of lung metastatic tumors produced by both SK-HEP-1 RNF214-sg2 and MHCC97H RNF214-sg2 cells more than Venus-∆CC (Fig. 7e, f). The data indicate that the CC domain is essential for RNF214 to facilitate the migration and metastasis of HCC.

## Discussion

In this study, our data showed that RNF214 exhibited elevated expression levels in HCC, which correlated with an unfavorable prognosis for patients. Moreover, RNF214 protein expression presented a positive correlation with serum AFP levels in patients suffering from HCC. Serum AFP is widely used as a biomarker for HCC screening and diagnosis, and HCC patients whose AFP level is below 20 ng/mL typically have a better prognosis [23]. AFP has the ability to accelerate the degradation of PTEN and trigger mTOR/AKT signaling, thereby stimulating the growth of HCC [24]. AFP also accelerates HCC development through inhibiting the HARH-mediated FSAD/FADD apoptosis pathway [25]. In addition, HCC samples with high AFP show alterations in immune-related pathways, such as the activation of TGF-β1 and IL-6 signaling pathways, the induction of SPP1 expression in macrophages, and the inhibition of T cell function [23, 26]. The positive correlation between RNF214 protein expression and the level of serum AFP in HCC patients indicates that RNF214 and AFP may regulate each other, which is worth investigating in the future.

Phase separation is critical for many biological processes, including regulation of the cellular stress response, maintenance of homeostasis, and cell development [27]. Dysregulation of physiological aggregates may contribute to disease development, and pathological aggregates can also activate abnormal signaling and transcriptional programs [28, 29]. Biomolecular aggregates formed by phase separation also have significant contributions to tumorigenesis. Phase separation has been found to influence transcriptional regulation, genomic stabilization, and signal transmission, thereby affecting tumor initiation and development [22]. Recently, several studies have explored the function of phase separation in HCC. CircVAMP3 inhibits the progression of HCC by inducing phase separation of CAPRIN1 to block the translation of c-Myc [30]. CircASH2 drives phase separation of YBX1 to increase TPM4 transcripts degradation and suppresses HCC metastasis by altering the tumor cytoskeleton structure [31]. LncRNA URB1-AS1 inhibits sorafenib-induced ferroptosis through promoting phase separation of ferritin in HCC [32]. The transcriptional complexes of Twist1, YY1, and p300 promote the malignancy of HCC by forming phase separation condensates at the super-enhancers of miR-9 to activate its expression, and metformin can disrupt these phase separation condensates to decrease miR-9 expression and the malignancy of HCC [33]. Glycogen accumulation leads to phase separation and promotes liver tumorigenesis, and the elimination of glycogen accumulation reduces the incidence of HCC [34]. Our present study revealed that RNF214 underwent phase separation, and the CC domain of RNF214 mediated its phase separation. Furthermore, the CC domain was necessary for the oncogenic function RNF214 in HCC. CC domain is a widespread and diverse structure that is found in ~10% of proteins, functioning as DNA-binding domains and protein–protein interaction domains participated in various biological processes [35]. Usually consisting of two or more α-helices that wrap around each other to form a supercoiled bundle, the CC domain is characterized by a repeating seven-residue long sequence pattern (abcdefg)_*n*_, where a and d are hydrophobic residues and others are hydrophilic residues [35]. It is reported that the CC domain drove the phase separation of several other proteins including LINE1 [36], MATN3 [37], TAZ [38], and YAP [39], etc. Therefore, our study reinforces the importance of the CC domain for phase separation.

Our experimental findings have revealed that RNF214 forms dimers through its CC domain, a discovery that sheds light on the role of the CC motif in the dimerization of RNF214 and its response to cellular conditions that induce phase separation. These insights are crucial for advancing our understanding of the function of RNF214 in cellular organization and signal transduction. While the RING finger domain is not directly implicated in phase separation, dimerization is frequently a requisite for activating the E3 ligase activity [40]. The phase separation mediated by the CC domain of RNF214 could be pivotal in activating the C-terminal ZnF/RING-dependent ligase activity, potentially playing a role in the regulation of HCC progression. However, the COSMIC database (https://cancer.sanger.ac.uk/cosmic) indicates that copy number variations and mutations within the CC and RING domains of the RNF214 gene are of low frequency in HCC. This suggests that mutations in these specific regions may not be the primary drivers of HCC. Nevertheless, other factors such as epigenetic modifications, alternative splicing, or post-translational modifications might play a more significant role in the pathogenesis of HCC associated with RNF214.

Recent studies have indicated that the use of FRAP and 1,6-hexanediol treatment to validate phase separation characteristics does not always distinguish phase separation from other scaffold-based assemblies [41]. Although FRAP is often used to demonstrate the liquid-like mobility of macromolecules within membraneless organelles, these analyses struggle to differentiate the interactions of phase separation from specific intermolecular interactions. Furthermore, 1,6-hexanediol is commonly used to demonstrate the dissolution of membraneless structures, thereby proving that phase separation is the driving force behind assembly, but it is toxic at high concentrations and exhibits significant polysemanticity. It must be acknowledged that there are certain limitations to using these methods alone in cells, while reconstituting phase separation in vitro is used to address this issue [42]. Therefore, our experiments were not only verified within cells but also through the purification of proteins in vitro to further validate and eliminate interference from other factors within the cell. Moreover, we conducted experiments under simulated physiological and pathological conditions for the RNF214 and CC domains as much as possible to ensure the reliability of the results. With the advancement of research techniques, we believe that more sophisticated technological means will become available to explore phase separation.

In conclusion, these results provided evidence that RNF214 was markedly upregulated in HCC and correlated with an unfavorable outcome. As an oncogene, RNF214 induced HCC proliferation, migration, and metastasis. Mechanically, RNF214 underwent phase separation and the CC domain of RNF214 mediated its phase separation. Furthermore, the CC domain was essential for the oncogenic activity of RNF214 in HCC. Our data suggest that phase separation of RNF214 contributes to the advancement of HCC. Additionally, RNF214 shows the promising potential as a biomarker and target for therapeutic intervention in HCC.

### Supplementary information

Supplementary Material

Original western blots

## Data Availability

The data generated and analyzed in this study are available from the corresponding authors upon request.

## References

[1] Zhao YG, Zhang H (2020). Phase separation in membrane biology: the interplay between membrane-bound organelles and membraneless condensates. Dev Cell.

[2] Xiao Q, McAtee CK, Su X (2022). Phase separation in immune signalling. Nat Rev Immunol.

[3] Banani SF, Lee HO, Hyman AA, Rosen MK (2017). Biomolecular condensates: organizers of cellular biochemistry. Nat Rev Mol Cell Biol.

[4] Mehta S, Zhang J (2022). Liquid-liquid phase separation drives cellular function and dysfunction in cancer. Nat Rev Cancer.

[5] Borcherds W, Bremer A, Borgia MB, Mittag T (2021). How do intrinsically disordered protein regions encode a driving force for liquid-liquid phase separation?. Curr Opin Struct Biol.

[6] Wang YL, Zhao WW, Shi J, Wan XB, Zheng J, Fan XJ (2023). Liquid-liquid phase separation in DNA double-strand breaks repair. Cell Death Dis.

[7] Lu J, Qian J, Xu Z, Yin S, Zhou L, Zheng S (2021). Emerging roles of liquid-liquid phase separation in cancer: from protein aggregation to immune-associated signaling. Front Cell Dev Biol.

[8] Llovet JM, Kelley RK, Villanueva A, Singal AG, Pikarsky E, Roayaie S (2021). Hepatocellular carcinoma. Nat Rev Dis Prim.

[9] Wang G, Wang Q, Liang N, Xue H, Yang T, Chen X (2020). Oncogenic driver genes and tumor microenvironment determine the type of liver cancer. Cell Death Dis.

[10] Chen S, Cao Q, Wen W, Wang H (2019). Targeted therapy for hepatocellular carcinoma: challenges and opportunities. Cancer Lett.

[11] Li S, Xue P, Diao X, Fan QY, Ye K, Tang XM (2023). Identification and validation of functional roles for three MYC-associated genes in hepatocellular carcinoma. J Adv Res.

[12] Dal Bo M, De Mattia E, Baboci L, Mezzalira S, Cecchin E, Assaraf YG (2020). New insights into the pharmacological, immunological, and CAR-T-cell approaches in the treatment of hepatocellular carcinoma. Drug Resist Updat.

[13] Barroso-Gomila O, Merino-Cacho L, Muratore V, Perez C, Taibi V, Maspero E (2023). BioE3 identifies specific substrates of ubiquitin E3 ligases. Nat Commun.

[14] Huang JR, Wang ST, Wei MN, Liu K, Fu JW, Xing ZH (2020). Piperlongumine alleviates mouse colitis and colitis-associated colorectal cancer. Front Pharmacol.

[15] Zhang W, Yang Y, Dong Z, Shi Z, Zhang JT (2019). Single-nucleotide polymorphisms in a short basic motif in the ABC transporter ABCG2 disable its trafficking out of endoplasmic reticulum and reduce cell resistance to anticancer drugs. J Biol Chem.

[16] Wang K, Xing ZH, Jiang QW, Yang Y, Huang JR, Yuan ML (2019). Targeting uPAR by CRISPR/Cas9 system attenuates cancer malignancy and multidrug resistance. Front Oncol.

[17] Huang SZ, Wei MN, Huang JR, Zhang ZJ, Zhang WJ, Jiang QW (2019). Targeting TF-AKT/ERK-EGFR pathway suppresses the growth of hepatocellular carcinoma. Front Oncol.

[18] Gao Q, Zhu H, Dong L, Shi W, Chen R, Song Z (2019). Integrated proteogenomic characterization of HBV-related hepatocellular carcinoma. Cell.

[19] Nozawa RS, Yamamoto T, Takahashi M, Tachiwana H, Maruyama R, Hirota T (2020). Nuclear microenvironment in cancer: control through liquid-liquid phase separation. Cancer Sci.

[20] Zhang H, Ji X, Li P, Liu C, Lou J, Wang Z (2020). Liquid-liquid phase separation in biology: mechanisms, physiological functions and human diseases. Sci China Life Sci.

[21] Tong X, Tang R, Xu J, Wang W, Zhao Y, Yu X (2022). Liquid-liquid phase separation in tumor biology. Signal Transduct Target Ther.

[22] Peng PH, Hsu KW, Wu KJ (2021). Liquid-liquid phase separation (LLPS) in cellular physiology and tumor biology. Am J Cancer Res.

[23] He H, Chen S, Fan Z, Dong Y, Wang Y, Li S (2023). Multi-dimensional single-cell characterization revealed suppressive immune microenvironment in AFP-positive hepatocellular carcinoma. Cell Discov.

[24] Wang S, Zhu M, Wang Q, Hou Y, Li L, Weng H (2018). Alpha-fetoprotein inhibits autophagy to promote malignant behaviour in hepatocellular carcinoma cells by activating PI3K/AKT/mTOR signalling. Cell Death Dis.

[25] Chen T, Dai X, Dai J, Ding C, Zhang Z, Lin Z (2020). AFP promotes HCC progression by suppressing the HuR-mediated Fas/FADD apoptotic pathway. Cell Death Dis.

[26] Munson PV, Adamik J, Butterfield LH (2022). Immunomodulatory impact of alpha-fetoprotein. Trends Immunol.

[27] Liu M, Li H, Luo X, Cai J, Chen T, Xie Y (2022). RPS: a comprehensive database of RNAs involved in liquid-liquid phase separation. Nucleic Acids Res.

[28] Conti BA, Oppikofer M (2022). Biomolecular condensates: new opportunities for drug discovery and RNA therapeutics. Trends Pharmacol Sci.

[29] Dolicka D, Foti M, Sobolewski C (2021). The emerging role of stress granules in hepatocellular carcinoma. Int J Mol Sci.

[30] Chen S, Cao X, Zhang J, Wu W, Zhang B, Zhao F (2022). circVAMP3 drives CAPRIN1 phase Separation and Inhibits hepatocellular carcinoma by suppressing c-Myc Translation. Adv Sci.

[31] Liu B, Shen H, He J, Jin B, Tian Y, Li W (2023). Cytoskeleton remodeling mediated by circRNA-YBX1 phase separation suppresses the metastasis of liver cancer. Proc Natl Acad Sci USA.

[32] Gao Y, Tong M, Wong TL, Ng KY, Xie YN, Wang Z (2023). Long noncoding RNA URB1-antisense RNA 1 (AS1) suppresses sorafenib-induced ferroptosis in hepatocellular carcinoma by driving ferritin phase separation. ACS Nano.

[33] Meng J, Han J, Wang X, Wu T, Zhang H, An H (2023). Twist1-YY1-p300 complex promotes the malignant progression of HCC through activation of miR-9 by forming phase-separated condensates at super-enhancers and relieved by metformin. Pharmacol Res.

[34] Liu Q, Li J, Zhang W, Xiao C, Zhang S, Nian C (2021). Glycogen accumulation and phase separation drives liver tumor initiation. Cell.

[35] Lupas AN, Gruber M (2005). The structure of alpha-helical coiled coils. Adv Protein Chem.

[36] Newton JC, Naik MT, Li GY, Murphy EL, Fawzi NL, Sedivy JM (2021). Phase separation of the LINE-1 ORF1 protein is mediated by the N-terminus and coiled-coil domain. Biophys J.

[37] Huang Y, Xu X, Lu Y, Sun Q, Zhang L, Shao J (2024). The phase separation of extracellular matrix protein matrilin-3 from cancer-associated fibroblasts contributes to gastric cancer invasion. FASEB J.

[38] Gerussi A, Luca M, Cristoferi L, Ronca V, Mancuso C, Milani C (2020). New therapeutic targets in autoimmune cholangiopathies. Front Med.

[39] Yu M, Peng Z, Qin M, Liu Y, Wang J, Zhang C (2021). Interferon-γ induces tumor resistance to anti-PD-1 immunotherapy by promoting YAP phase separation. Mol Cell.

[40] Fiorentini F, Esposito D, Rittinger K (2020). Does it take two to tango? RING domain self-association and activity in TRIM E3 ubiquitin ligases. Biochem Soc Trans.

[41] Muzzopappa F, Hummert J, Anfossi M, Tashev SA, Herten DP, Erdel F (2022). Detecting and quantifying liquid-liquid phase separation in living cells by model-free calibrated half-bleaching. Nat Commun.

[42] Hedtfeld M, Dammers A, Koerner C, Musacchio A (2024). A validation strategy to assess the role of phase separation as a determinant of macromolecular localization. Mol Cell.

