# Supplementary material for: Phase separation of RNF214 promotes the progression of hepatocellular carcinoma
Source: Cell Death Dis. 2024 Jul 5;15(7):483. doi: 10.1038/s41419-024-06869-2 (PMC11226663; doi:10.1038/s41419-024-06869-2)
Supplement: Supplementary file 1 — Supplementary Material [file 41419_2024_6869_MOESM1_ESM.pdf]

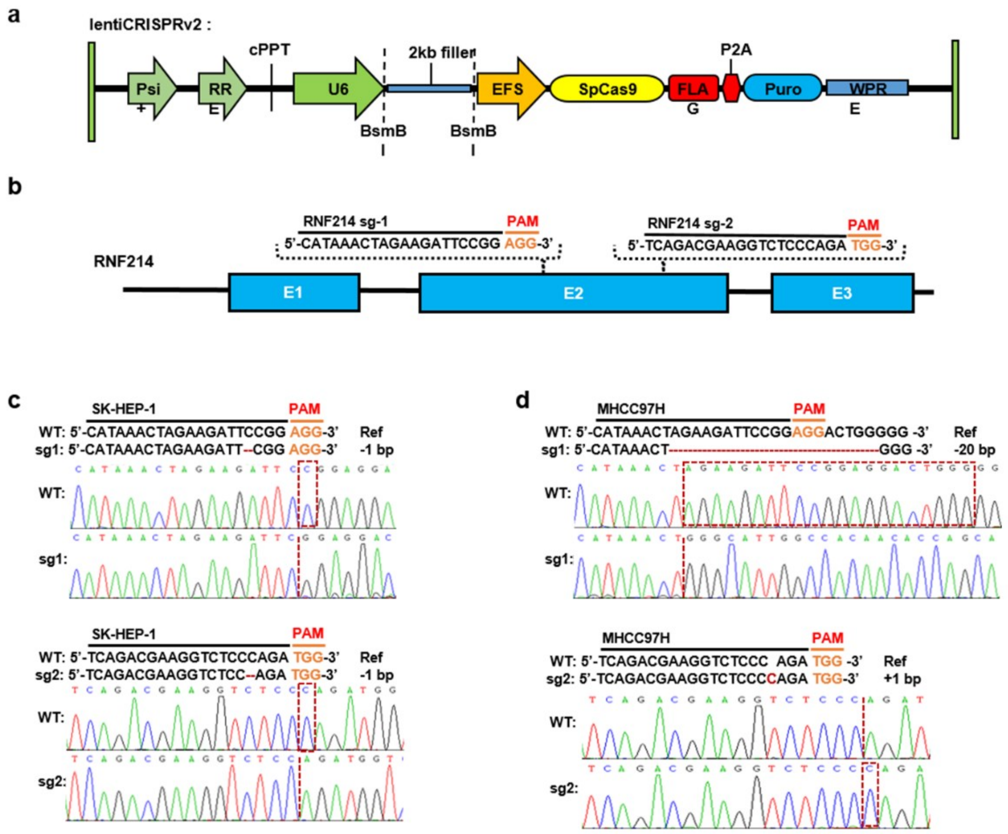

Supplemental Figure S1 HCC cells with knockout of RNF214 were established. Related to Fig. 2. (a) The schematic diagram of lentiCRISPR V2 was shown. (b) Two sgRNA sequences on the exon2 of RNF214 gene were designed. (c, d) The genome sequences of SK-HEP-1 and MHCC97H cells with knockout of RNF214 were shown.

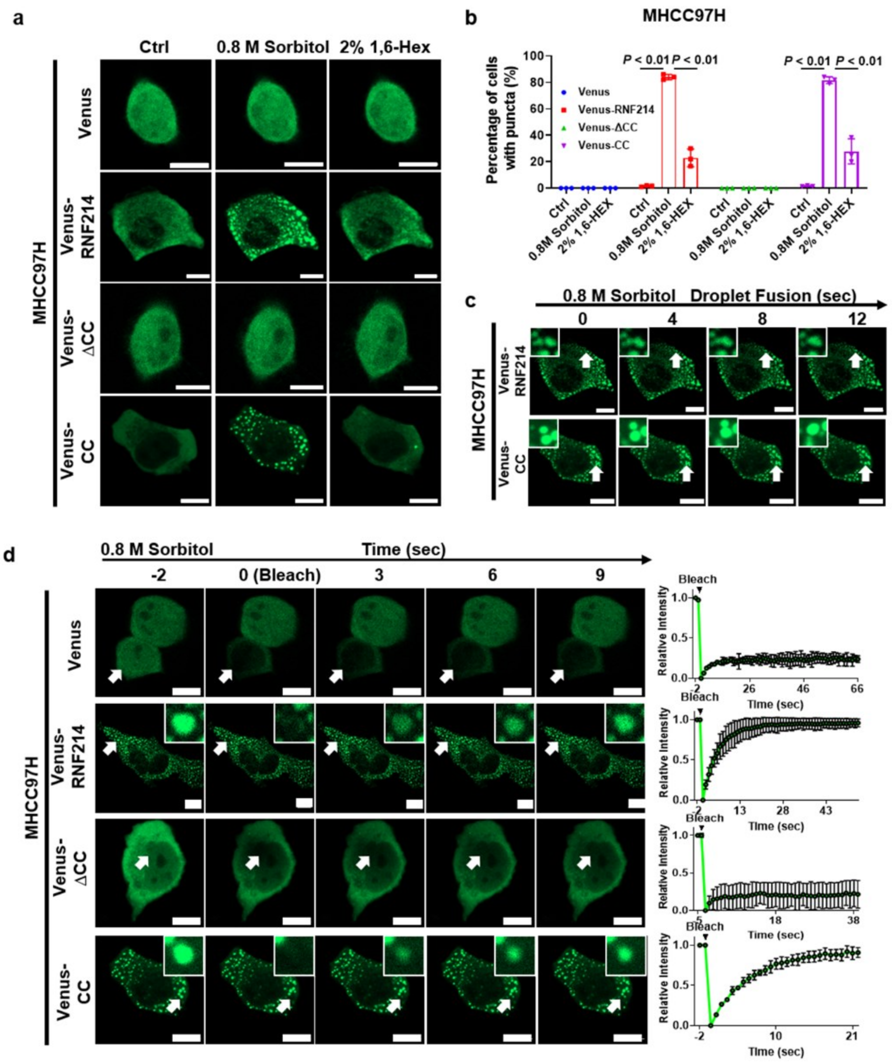

Supplemental Figure S2 The CC domain of RNF214 mediated its phase separation in HCC cells treated with 0.8 M sorbitol for 10 minutes. Related to Fig. 5. (a, b) MHCC97H cells transfected with Venus, Venus-RNF214 full-length and truncations were observed and quantified after being treated with 0.8 M sorbitol for 10 minutes and 2% 1,6-hexadiol for another 3 minutes. (c) Observation of the puncta fusion. (d) Observation of the puncta after the fluorescence bleaching. Scale bar = 10 μm. Data was presented as mean ± SD. Statistical significance was set at $p < 0.05$.

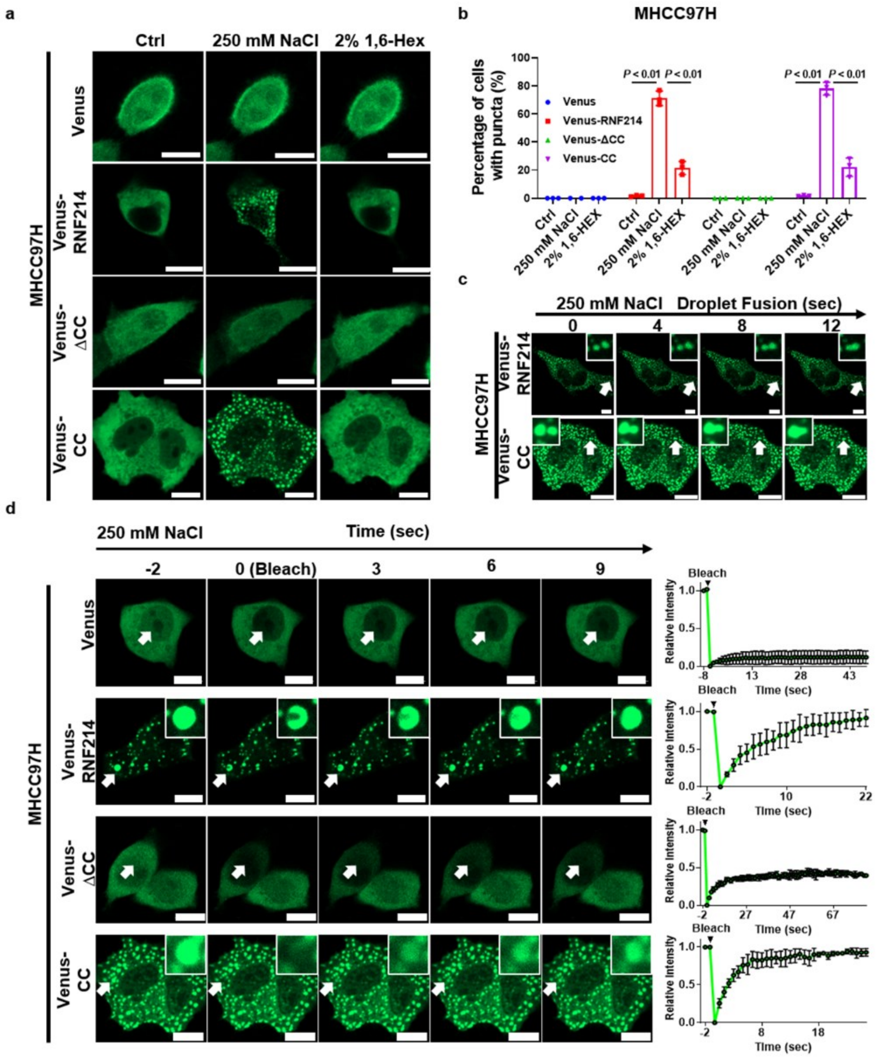

Supplemental Figure S3 The CC domain of RNF214 mediated its phase separation in HCC cells treated with 250 mM NaCl for 10 minutes. Related to Fig. 5. (a, b) MHCC97H cells transfected with Venus, Venus-RNF214 full-length and truncations were observed and quantified after being treated with 250 mM NaCl for 10 minutes and 2% 1,6-hexadiol for another 3 minutes. (c) Observation of the puncta fusion. (d) Observation of the puncta after the fluorescence bleaching. Scale bar = 10 μm. Data was presented as mean ± SD. Statistical significance was set at $p < 0.05$.

Supplemental Table S1 The correlation between RNF214 expression and clinicopathological characteristics of 150 HBV-Related HCC patients.

Characteristics	RNF214 Low	RNF214 High	*P* Value
N	86	64	
**Gender, N (%)**			0.053
Male	74 (49.3%)	47 (31.3%)	
Female	12 (8%)	17 (11.3%)	
**Age, N (%)**			**0.027***
<53	34 (22.7%)	37 (24.7%)	
≥53	52 (34.7%)	27 (18%)	
**BCLC stage, N (%)**			0.318
A	42 (28%)	26 (17.3%)	
B+C	44 (29.3%)	38 (25.3%)	
**TNM stage, N (%)**			0.985
I+II	59 (39.3%)	44 (29.3%)	
III+IV	27 (18%)	20 (13.3%)	
**Liver cirrhosis, N (%)**			0.666
No	27 (18%)	18 (12%)	
Yes	59 (39.3%)	46 (30.7%)	
**Tumor number, N (%)**			0.223
1	61 (40.7%)	51 (34%)	
≥2	25 (16.7%)	13 (8.7%)	
**Tumor size (cm), N (%)**			0.247
<5	39 (26%)	23 (15.3%)	
≥5	47 (31.3%)	41 (27.3%)	
**PT, (s), N (%)**			0.372
<14	81 (54%)	63 (42%)	
≥14	5 (3.3%)	1 (0.7%)	
**ALT, (U/L), N (%)**			0.918
<40	49 (32.7%)	37 (24.7%)	
≥40	37 (24.7%)	27 (18%)	
**ALB, (g/L), N (%)**			0.216
<35	4 (2.7%)	0 (0%)	
≥35	82 (54.7%)	64 (42.7%)	
**γ-GT, (U/L), N (%)**			0.879
<50	36 (24%)	26 (17.3%)	
≥50	50 (33.3%)	38 (25.3%)	
**AFP（ng/mL）, N (%)**			**0.032***
<20	39 (26%)	18 (12%)	
≥20	47 (31.3%)	46 (30.7%)	
**Recurrence, n (%)**			0.57
No	43 (28.7%)	35 (23.3%)	
Yes	43 (28.7%)	29 (19.3%)	

Notes: *P < 0.05 was considered significant.

Supplemental Table S2 The univariate and multivariate Cox regression analyses of risk factors associated with overall survival.

**Characteristics**	**Univariate analysis**		**Multivariate analysis**	
	**Hazard ratio (95% CI)**	***P* Value**	**Hazard ratio (95% CI)**	***P* Value**
**Gender**				
Male	Reference			
Female	1.387 (0.743 - 2.588)	0.304		
**Age**				
<53	Reference		Reference	
≥53	0.521 (0.304 - 0.894)	**0.018***	0.744 (0.427 - 1.297)	0.297
**BCLC stage**				
A	Reference			
B+C	1.054 (0.618 - 1.798)	0.846		
**TNM stage**				
I+II	Reference			
III+IV	0.767 (0.423 - 1.388)	0.38		
**Liver cirrhosis**				
No	Reference		Reference	
Yes	2.556 (1.249 - 5.231)	**0.010***	2.039 (0.991 - 4.199)	0.053
**Tumor number**				
1	Reference			
≥2	1.165 (0.651 - 2.084)	0.608		
**Tumor size (cm)**				
<5	Reference			
≥5	1.160 (0.673 - 1.999)	0.593		
**PT (s)**				
<14	Reference			
≥14	0.688 (0.167 - 2.829)	0.604		
**ALT (U/L)**				
<40	Reference			
≥40	1.290 (0.760 - 2.189)	0.346		
**ALB (g/L)**				
<35	Reference			
≥35	2.343 (0.730 - 7.521)	0.152		
**γ-GT (U/L)**				
<50	Reference			
≥50	1.095 (0.635 - 1.886)	0.744		
**AFP（ng/mL）**				
<20	Reference		Reference	
≥20	2.614 (1.378 - 4.961)	**0.003***	2.572 (1.344 - 4.922)	**0.004***
**Recurrence**				
No	Reference		Reference	
Yes	3.170 (1.769 - 5.681)	**< 0.001***	3.263 (1.780 - 5.982)	**< 0.001***
**RNF214**				
Low	Reference		Reference	
High	1.818 (1.068 - 3.093)	**0.028***	1.873 (1.084 - 3.235)	**0.024***

Notes: *$P < 0.05$ was considered significant
