# Supplementary material for: Phase separation of RNF214 promotes the progression of hepatocellular carcinoma
Source: Cell Death Dis. 2024 Jul 5;15(7):483. doi: 10.1038/s41419-024-06869-2 (PMC11226663; doi:10.1038/s41419-024-06869-2)
Supplement: Supplementary file 2 — Original western blots [file 41419_2024_6869_MOESM2_ESM.pptx]

## Slide 1
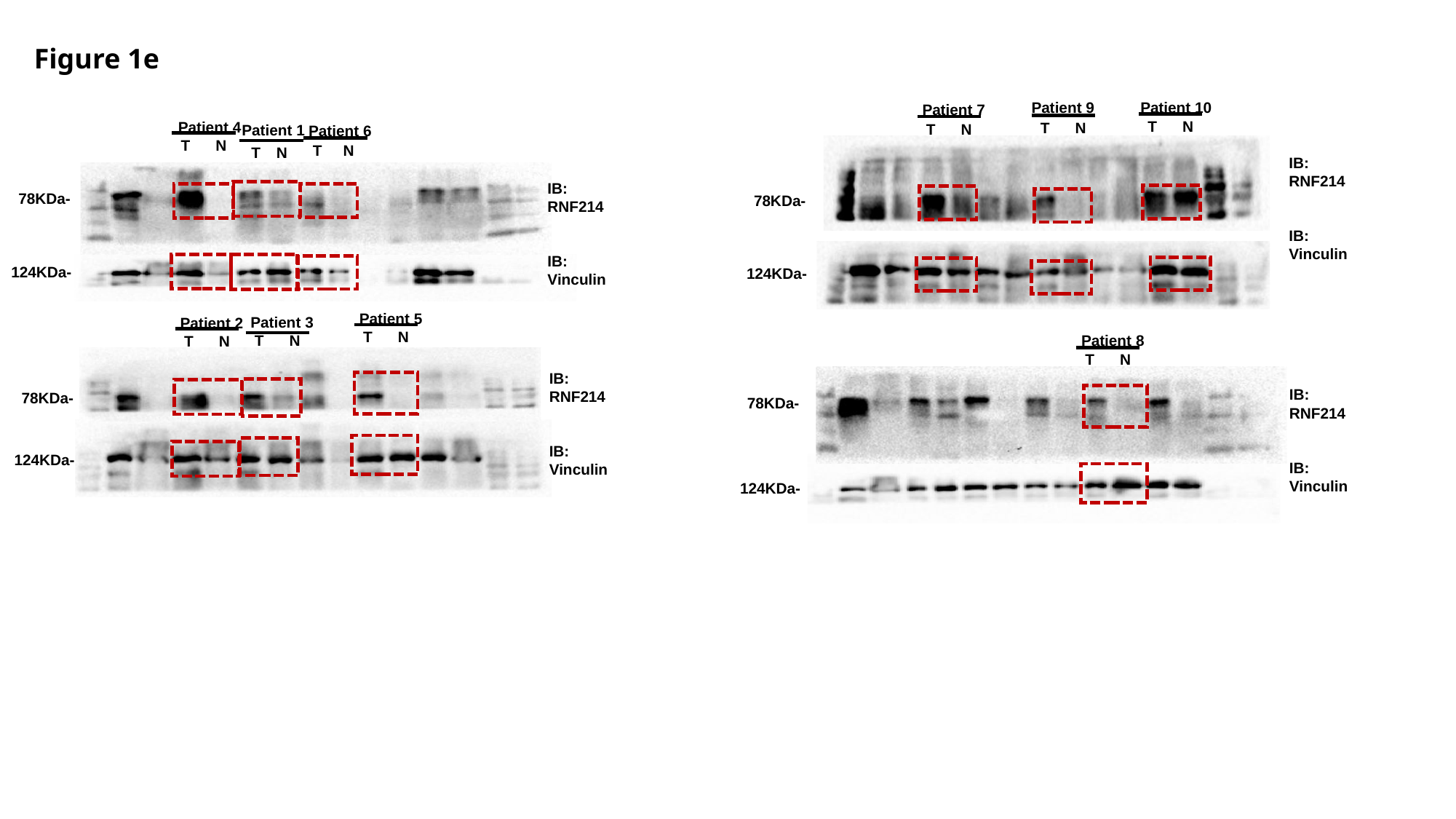

Figure 1e
Patient 10
Patient 9
Patient 7
T
N
Patient 4
T
N
T
N
Patient 1
Patient 6
T
N
T
N
T
N
IB:
RNF214
IB:
RNF214
78KDa-
78KDa-
IB:
Vinculin
IB:
Vinculin
124KDa-
124KDa-
Patient 5
Patient 3
Patient 2
T
N
Patient 8
T
N
T
N
T
N
IB:
RNF214
IB:
RNF214
78KDa-
78KDa-
IB:
Vinculin
124KDa-
IB:
Vinculin
124KDa-

## Slide 2
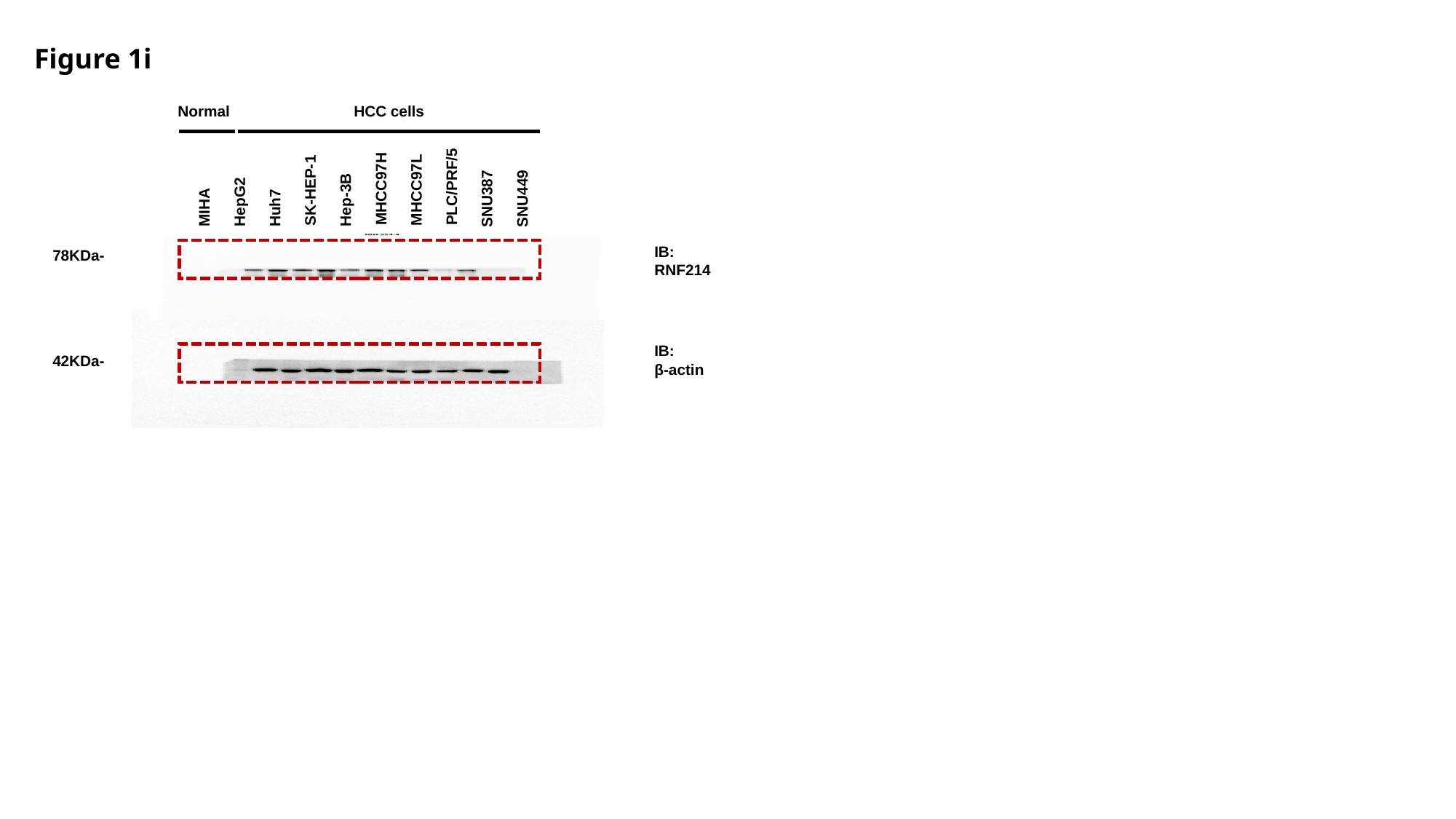

Figure 1i
HCC cells
Normal
PLC/PRF/5
MHCC97H
MHCC97L
SK-HEP-1
SNU449
HepG2
SNU387
Hep-3B
MIHA
Huh7
IB:
RNF214
78KDa-
IB:
β-actin
42KDa-

## Slide 3
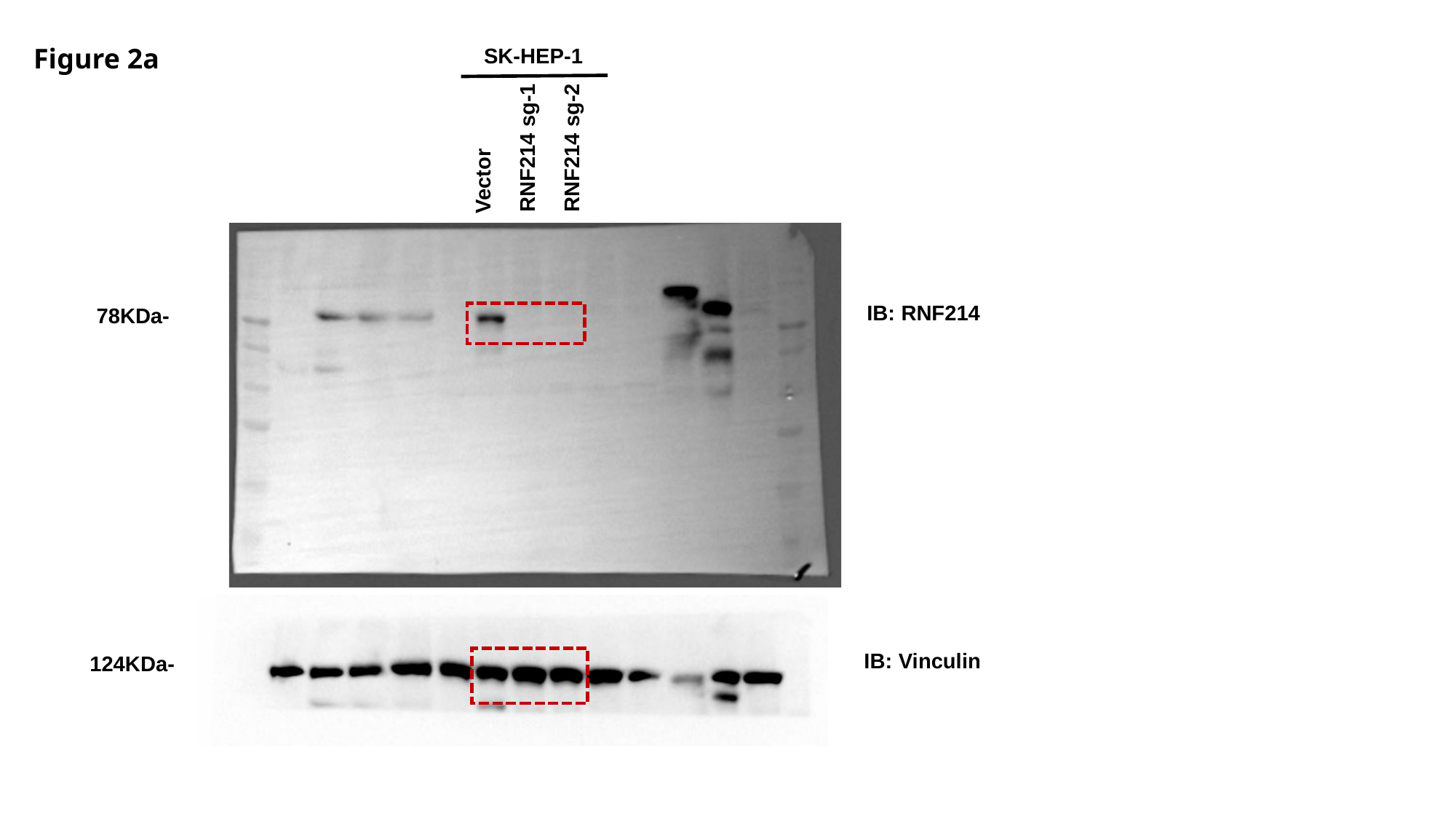

Figure 2a
RNF214 sg-1
SK-HEP-1
RNF214 sg-2
Vector
IB: RNF214
78KDa-
IB: Vinculin
124KDa-

## Slide 4
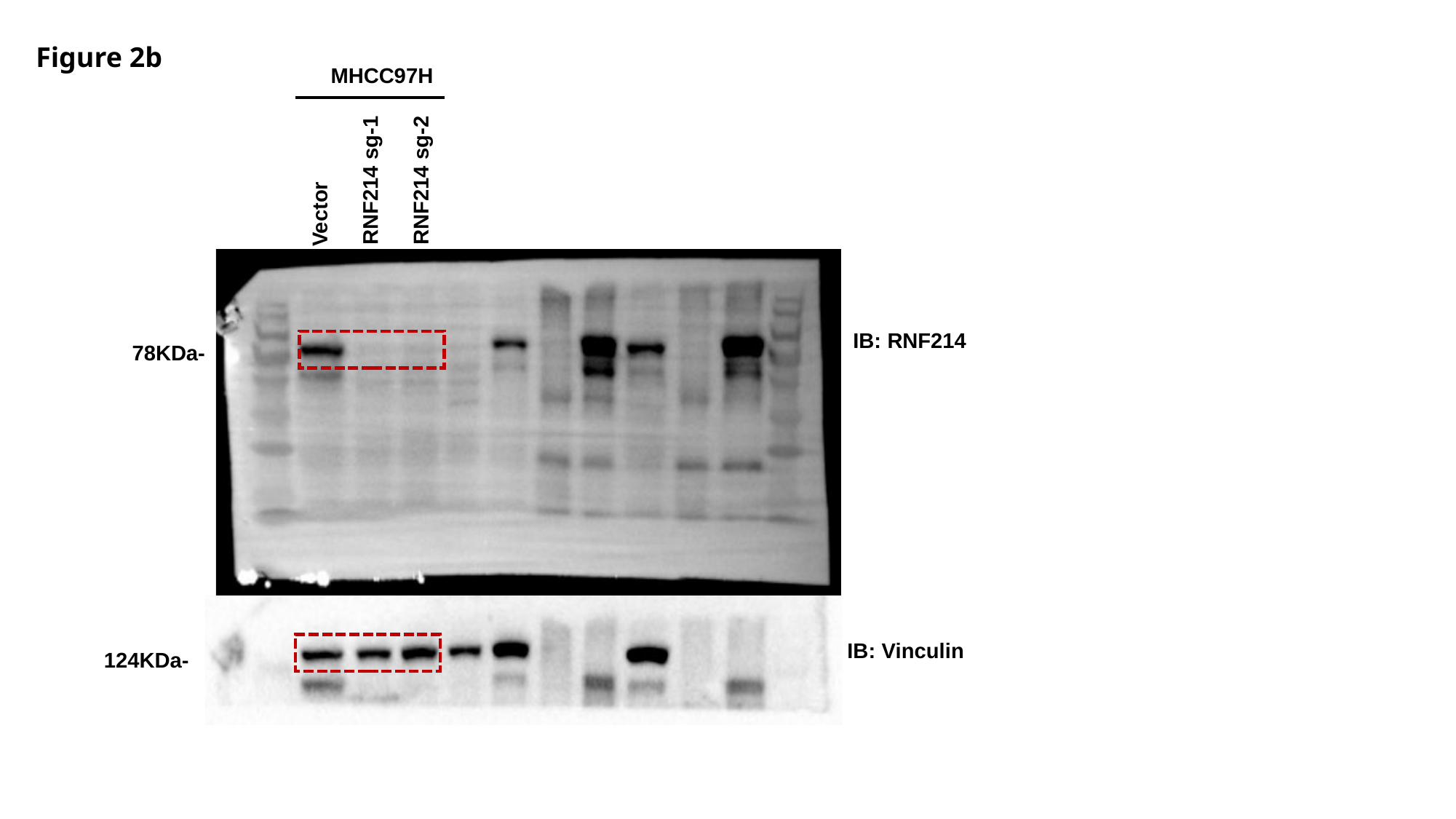

Figure 2b
MHCC97H
RNF214 sg-1
RNF214 sg-2
Vector
IB: RNF214
78KDa-
IB: Vinculin
124KDa-

## Slide 5
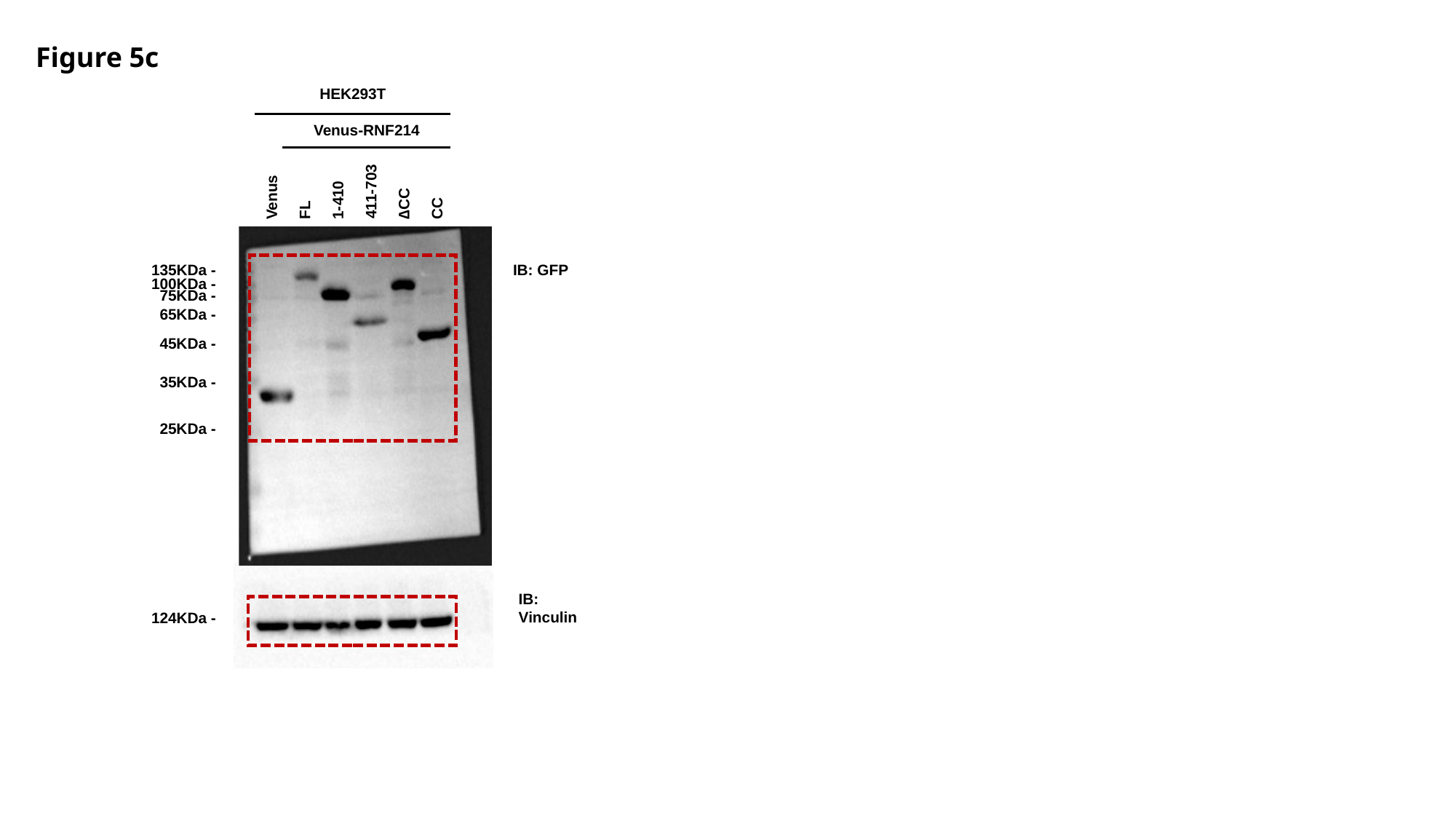

c
Figure 5c
HEK293T
Venus-RNF214
Venus
411-703
1-410
∆CC
CC
FL
IB: GFP
135KDa -
100KDa -
75KDa -
65KDa -
45KDa -
35KDa -
25KDa -
IB:
Vinculin
124KDa -

## Slide 6
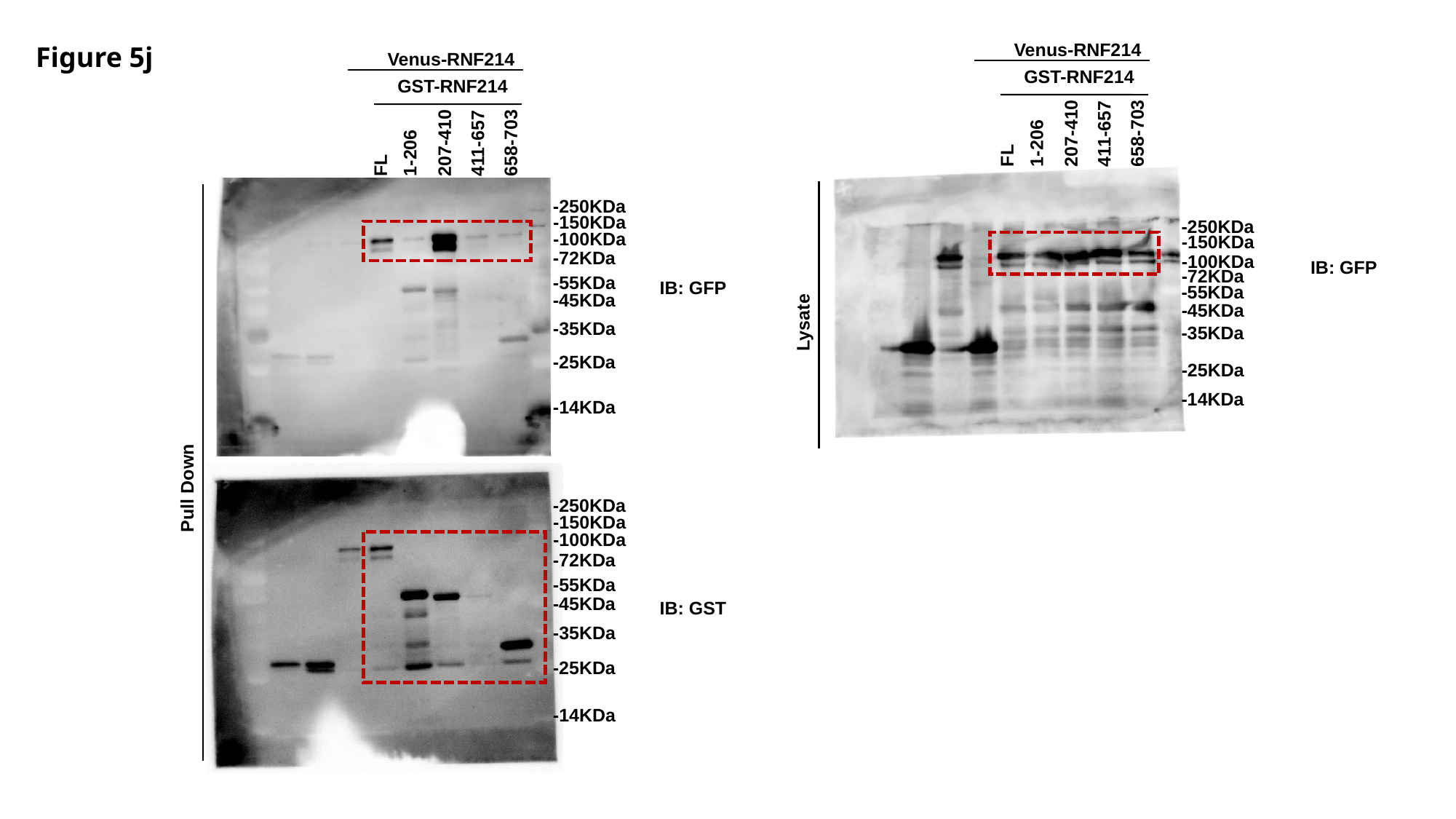

Venus-RNF214
Figure 5j
Venus-RNF214
GST-RNF214
411-657
207-410
658-703
1-206
FL
-250KDa
-150KDa
-100KDa
-72KDa
-55KDa
-45KDa
-35KDa
-25KDa
-14KDa
IB: GFP
Pull Down
-250KDa
-150KDa
-100KDa
-72KDa
-55KDa
-45KDa
-35KDa
-25KDa
-14KDa
IB: GST
GST-RNF214
411-657
207-410
658-703
1-206
FL
-250KDa
-150KDa
-100KDa
-72KDa
-55KDa
-45KDa
-35KDa
-25KDa
-14KDa
IB: GFP
Lysate

## Slide 7
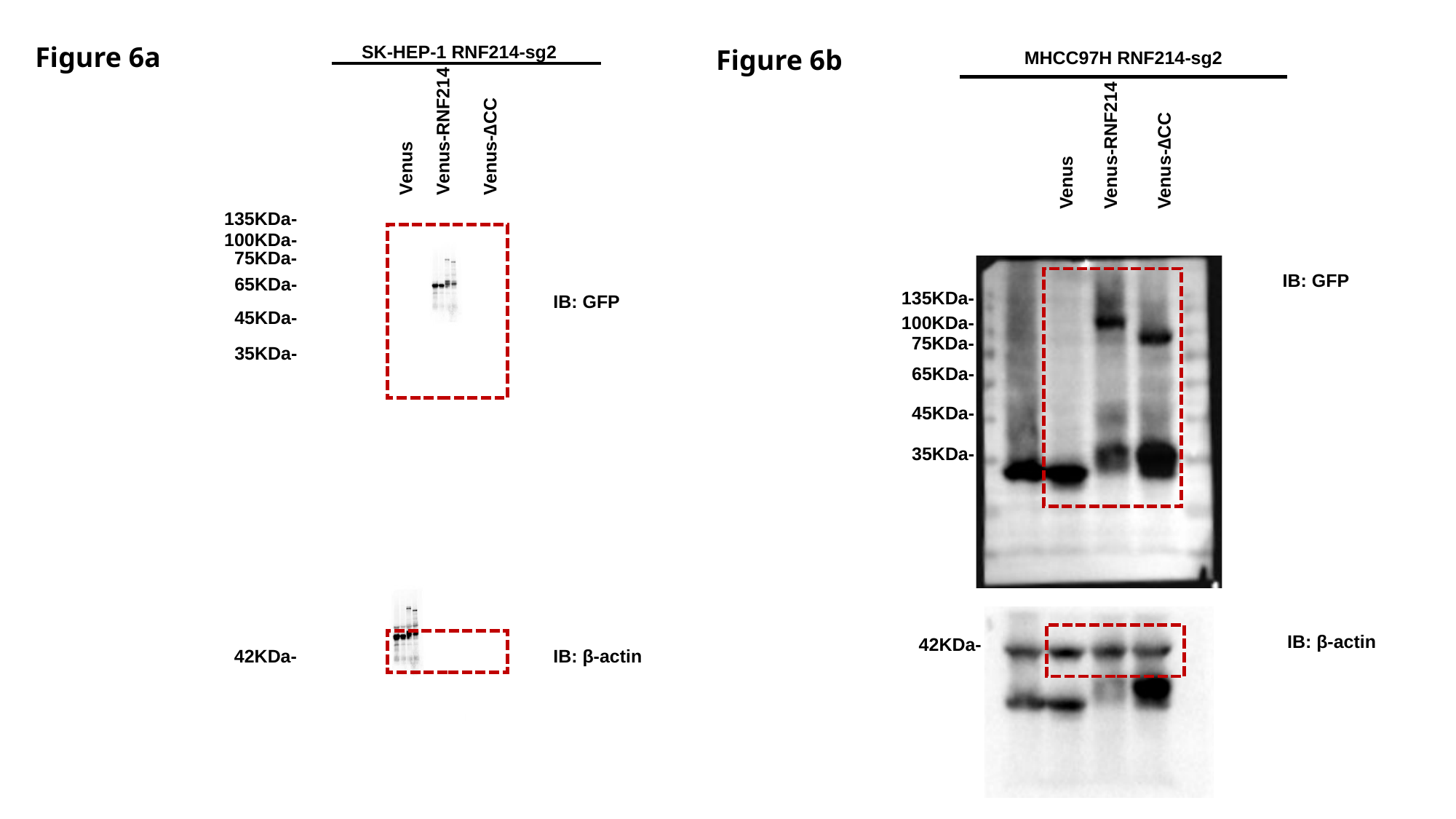

Venus-RNF214
Venus-∆CC
SK-HEP-1 RNF214-sg2
Figure 6a
Figure 6b
MHCC97H RNF214-sg2
Venus-∆CC
Venus-RNF214
Venus
Venus
135KDa-
100KDa-
75KDa-
65KDa-
IB: GFP
45KDa-
35KDa-
IB: GFP
135KDa-
100KDa-
75KDa-
65KDa-
45KDa-
35KDa-
42KDa-
IB: β-actin
IB: β-actin
42KDa-
